# A novel gene LbHLH from the halophyte *Limonium bicolor* enhances salt tolerance via reducing root hair development and enhancing osmotic resistance

**DOI:** 10.1186/s12870-021-03094-3

**Published:** 2021-06-22

**Authors:** Xi Wang, Yingli Zhou, Yanyu Xu, Baoshan Wang, Fang Yuan

**Affiliations:** Shandong Provincial Key Laboratory of Plant Stress, College of Life Sciences, Shandong Normal University, Ji’nan, Shandong P.R. China

**Keywords:** Heterologous expression, *Limonium bicolor*, Osmotic stress, Root hair, Trichome, Salt resistance

## Abstract

**Background:**

Identifying genes involved in salt tolerance in the recretohalophyte *Limonium bicolor* could facilitate the breeding of crops with enhanced salt tolerance. Here we cloned the previously uncharacterized gene *LbHLH* and explored its role in salt tolerance.

**Results:**

The 2,067-bp open reading frame of *LbHLH* encodes a 688-amino-acid protein with a typical helix-loop-helix (HLH) domain. In situ hybridization showed that *LbHLH* is expressed in salt glands of *L. bicolor*. LbHLH localizes to the nucleus, and *LbHLH* is highly expressed during salt gland development and in response to NaCl treatment. To further explore its function, we heterologously expressed *LbHLH* in *Arabidopsis thaliana* under the 35S promoter. The overexpression lines showed significantly increased trichome number and reduced root hair number. LbHLH might interact with GLABRA1 to influence trichome and root hair development, as revealed by yeast two-hybrid analysis. The transgenic lines showed higher germination percentages and longer roots than the wild type under NaCl treatment. Analysis of seedlings grown on medium containing sorbitol with the same osmotic pressure as 100 mM NaCl demonstrated that overexpressing *LbHLH* enhanced osmotic resistance.

**Conclusion:**

These results indicate that LbHLH enhances salt tolerance by reducing root hair development and enhancing osmotic resistance under NaCl stress.

**Supplementary Information:**

The online version contains supplementary material available at 10.1186/s12870-021-03094-3.

## Background

According to the Food and Agriculture Organization of the United Nations (FAO), soil salinity affects 950 million hectares of land worldwide, accounting for more than 6.5% of the world’s total land area [1, 2]. Nearly half of the irrigated soil worldwide is affected by salinization [3]. Increasing salinization reduces the arable land area, which seriously affects food production and food security [1, 4, 5]. Therefore, it is urgent to design ways to develop and utilize saline lands [6, 7]. Traditional saline soil improvement methods, such as fresh water pressure and concealed pipe discharge, are costly and lead to secondary soil salinization [8]. Hence, it is crucial to develop biological methods to improve saline lands [9].

Halophytes grow normally and complete their lifecycles in saline habitats containing ≥ 200 mM NaCl [4]. Halophytes are divided into three categories based on the mechanisms they use for ion accumulation and transport: (1) euhalophytes (dilute halophytes), such as *Suaeda salsa*, sequester salt ions in the vacuole [10]; (2) pseudohalophytes (salt-repellent halophytes), such as *Phragmites communis*, block salt from entering cells [11]; and (3) recretohalophytes, such as *Limonium bicolor* and *Mesembryanthemum crystallinum*, secrete salt to the outside environment [12]. Unlike other halophytes, recretohalophytes have specialized structures, including salt bladders and salt glands [13], that collect or secrete salt out of the plant to avoid salt stress [14]. Bladders accumulate salt, whereas salt glands actively excrete excess salt out of the plant [15].

*L. bicolor* is a typical recretohalophyte, with salt glands on its stems and leaves that excrete excess salt ions [16, 17]. *L. bicolor* is considered to be a pioneer plant for improving saline soils. It is easy to observe salt glands in this plant under a fluorescence microscope, as they exhibit blue autofluorescence [18]. The development of the first true leaves of *L. bicolor* can be divided into five stages: the undifferentiated stage (A), the salt gland differentiation stage (B), the stomata differentiation stage (C), the pavement cell differentiation stage (D), and the mature stage (E) [19].

Transcriptome profiling of leaves at these stages has uncovered various candidate genes involved in salt gland differentiation [13, 19, 20]. Some of these genes are highly homologous to genes related to trichome development in other plants, such as *GLABRA1* (*GL1*), *TRANSPARENT TESTA GLABRA1* (*TTG1*), *GLABRA3* (*GL3*), *ENHANCER OF GLABRA 3* (*EGL3*), *SUPER SENSITIVE TO ABA AND DROUGHT2* (*SAD2*), *TRIPTYCHON* (*TRY*), and *CAPRICE* (*CPC*) [19]. The functions of some of these genes have been demonstrated via heterologous expression in *Arabidopsis thaliana.* For example, the heterologous expression of *LbTTG1* or *LbSAD2* increased trichome development and salt resistance in Arabidopsis [21, 22], whereas expression of *LbTRY* increased root hair development and salt sensitivity [23]. These findings suggest that salt glands and trichomes could be homologous organs arising from the same ancestor.

These transcriptome studies also uncovered another group of candidate genes of uncharacterized/unknown function that are highly expressed during different stages of salt gland development. Given that no plants with publicly available genome sequences have salt glands, these un-annotated genes are thought to be unique to salt glands and may play important roles in salt gland development.

In the current study, we investigated the role of *Lb1G04899*, a gene of unknown function that is highly expressed in *L. bicolor* during early salt gland development, as determined by transcriptome analysis [19]. No other family members of this gene were detected, and it only produces one type of transcript. Since *Lb1G04899* encodes a protein with a typical helix-loop-helix domain, we named this gene *LbHLH*. Arabidopsis plants heterologously expressing this gene showed increased trichome development, reduced root hair development, and enhanced salt tolerance. LbHLH might interact with AtGL1, as revealed in a yeast two-hybrid assay. We propose a possible mechanism for the roles of this previously uncharacterized gene in the differentiation of both trichomes and root hairs as well as in salt resistance.

## Methods

### Plant materials and growth conditions

*Limonium bicolor* seeds were obtained from plants grown in saline soil (N37°20'; E118°36') in the Yellow River Delta (Shandong Province, China) with the permission of the Dongying government. The author Baoshan Wang had formally identified *L. bicolor*, and the seeds harvesting process is in full compliance with relevant government guidelines. Unfortunately, we were unable to find a voucher specimen of *L. bicolor* stored in any publicly available herbarium. The dried seeds were stored at 4 °C for at least six months [24]. Before use, the seeds were surface-disinfected with 70% ethanol (5 min), followed by 6% (v/v) NaClO (Sigma, USA) for 17 min with shaking. The seeds were washed five times with sterile distilled water and sown on MS basal medium. The plants were cultured at 28 ± 3 °C/23 ± 3 °C (day/night) at a light intensity of 600 μmol/m^2^/s (15 h photoperiod) and 65% relative humidity.

The *Arabidopsis thaliana* ecotype Col-0 (Columbia-0) seeds used are originally preserved in our laboratory. The author Baoshan Wang had formally identified Col-0 before. The collection of seeds and the performance of experimental research on such plant were complied with the national guidelines of China. *Arabidopsis thaliana* ecotype Col-0 (Columbia-0) seeds were sterilized three times with 70% alcohol for four minutes each time and three times with 95% alcohol for four minutes each time. The sterilized seeds were washed with sterile distilled water and sown on 1/2MS medium. After two days of stratification at 4 °C, the plants were cultivated at 22 °C/18 °C (day/night) under a 16 h/8 h light/dark cycle with a light level of 150 μmol/m^2^/s and 70% relative humidity [25]. To facilitate infection and transformation by *Agrobacterium tumefaciens*, seedlings were cultivated for one week on 1/2MS medium and transplanted into pots (9 cm height × 9 cm diameter) filled with nutrient-rich soil (soil:vermiculite:perlite, 3:1:1).

### Cloning and bioinformatic analysis of *LbHLH*

The first true leaves of *L. bicolor* were collected at different stages of leaf development, including the undifferentiated stage (stage A; 4–5 days after sowing, 5000 leaves), salt gland differentiation stage (stage B; 6–7 days after sowing, 4000 leaves), stomata differentiation stage (stage C; 8–10 days after sowing, 3000 leaves), pavement cell differentiation stage (stage D; 11–16 days after sowing, 1000 leaves), and mature stage (stage E; more than 17 days after sowing, 1000 leaves) according to Yuan [19].

The total RNA was extracted from pooled leaves of each stage using a FastPure Plant Total RNA Isolation kit (RC401-01; Vazyme Biotech Co., Ltd.). cDNA was reverse transcribed from the RNA with a ReverTra Ace quantitative PCR (qPCR) RT kit (TOYOBO Co., Ltd, Japan) according to the manufacturer’s instructions. The reference sequence of *LbHLH* was obtained from the assembled sequence from a previously reported transcriptome [19]. Full-length *LbHLH* was cloned using the primers *LbHLH*-S and *LbHLH*-A (Table S1), which were designed with Primer Premier 5.0. The conserved domain of *LbHLH* was predicted using the online tool SMART (http://smart.embl-heidelberg.de/).

### Subcellular localization of LbHLH

The subcellular localization of LbHLH was determined using transformed onion epidermal cells harboring a GFP expression vector [26]. The pCAMBIA1300 vector was digested with SalI to form a linear vector. To obtain *LbHLH* cDNA, primers *LbHLH* OE-S and *LbHLH* OE-A were designed with SalI digestion sites (Table S1). The full-length coding sequence (CDS) of *LbHLH* carrying a SalI digestion site was introduced into the pCAMBIA1300 vector under the control of the CaMV 35S promoter by homologous recombination using a ClonExpress II One Step Cloning Kit (Vazyme Biotech Co., Ltd., China). *Agrobacterium tumefaciens* GV3101 was used to transform the pCAMBIA1300-*LbHLH* recombinant vector into onion epidermal cells [27]. After two days of cultivation in the light, fluorescent signals of GFP-labeled LbHLH were detected under a TCS S8 MP two-photon laser-scanning confocal microscope (Leica, Germany). DAPI was used to locate the nucleus and was observed under excitation at 358 nm [28]. FM4 − 64 (N-(3-Triethylammoniumpropyl)-4-(6-(4-(Diethylamino)Phenyl) Hexatrienyl) Pyridinium Dibromide, Invitrogen) was used to locate the plasma membrane and observed under excitation at 559 nm [29].

### Expression analysis and in situ hybridization of *L. bicolor*

A-E stage leaves, stems, roots, and aged leaves of *L. bicolor* grown on MS medium were collected for RNA extraction. Seedlings grown under different treatments (100 mM NaCl, 0.04 mg/L 6-BA (6-Benzylaminopurine) and 0.1 mg/L ABA (abscisic acid) added in MS medium) were also collected for RNA extraction. Quantitative RT-PCR primers *LbHLH*-RT-S and *LbHLH*-RT-A were designed using Beacon Designer software (version 7.8) (Table S1). RT-PCR was performed in a 20 μl reaction system including 10 μl SYBR qPCR Master Mix (Vazyme Biotech Co., Ltd.), 0.2 μM primers, and 300 ng cDNA in a fluorometric thermal cycler (Bio-Rad CFX96 ™ Realtime PCR System) under the following conditions: 95 °C for 30 s, 40 cycles (95 °C for 5 s, 60 °C for 30 s). *Lbtubulin* (primers *Lbtubulin*-RT-S and *Lbtubulin*-RT-A, Table S1) was used as an internal control [18]. The expression level of *LbHLH* in different tissues was calculated relative to the expression level in roots (which was set to 1)*.* Three biological replicates (separate experiments) were performed. Relative expression levels were calculated using the formula 2^–ΔΔC(T)^.

To further explore the expression patterns of *LbHLH* in *L. bicolor*, developing leaves (the first true leaf at 5–8 days of germination) were isolated from *L. bicolor* for in situ hybridization. Briefly, the leaves were fixed in 4% paraformaldehyde, embedded in paraffin, and dehydrated through an alcohol series. Thin Sects. (8 μm) of tissue were treated with proteinase K and hybridized in 6 ng/μL hybridization solution at 37 °C overnight. Digoxin-labeled *LbHLH* probe (5’-DIG-CUCCCUAACAUUAACCUUCAGAUCCAGCCC-3’, purified by HPLC) appeared blue-violet.

### Cloning of the *LbHLH* promoter and histochemical analysis

Genomic DNA was extracted from *L. bicolor* using a FastPure Plant DNA Isolation Mini Kit (Vazyme Biotech Co., Ltd.) to obtain the full-length *LbHLH* promoter. The reference sequence of the *LbHLH* promoter was obtained from the *L. bicolor* genome (unpublished) and the about 2-kb target sequence upstream of the start codon was retained to be considered the promoter sequence for reference. The promoter sequence was cloned using primers *LbHLH*-P-S and *LbHLH*-P-A (Table S1). Elements in the promoter were predicted using PlantCARE (http://bioinformatics.psb.ugent.be/webtools/plantcare/html/), and maps were drawn using CSDS 2.0 ( http://gsds.gao-lab.org/).

To replace the 35S promoter with the *LbHLH* promoter in pCAMBIA3301, HindIII and NcoI were used to excise the CaMV 35S promoter from pCAMBIA3301 and to obtain a linear vector. The promoter was cloned into the vector using primers 3301-*LbHLH*-P-S and 3301-*LbHLH*-P-A (Table S1) to add HindIII and NcoI digestion sites in advance. The linear vector pCAMBIA3301 and the inserted *LbHLH* promoter were ligated together using an In-Fusion HD Cloning Kit (Takara) to construct the recombinant vector.

*Agrobacterium tumefaciens* GV3101 cells were transformed with the recombinant plasmid and used to infect *Arabidopsis thaliana* Col-0 to generate Col::*pLbHLH*-GUS. The transgenic seedlings were continuously screened with herbicides (0.1%, v/v), and homozygous plants of the T3 generation were subjected to histochemical staining. Ten-day-old seedlings were immersed in GUS staining solution and incubated at 37 °C overnight with shaking. The stained plant materials were decolorized by incubating in 70% ethanol 2–3 times and observed under a dissecting microscope (Nikon, Japan)[30].

### Generation of Col-35*S::LbHLH* plants

The full-length CDS of *LbHLH* was cloned using primers *LbHLH* OEAt-S and *LbHLH* OEAt-A (Table S1) containing NcoI digestion sites for cloning into different vectors. A ClonExpress II One Step Cloning Kit (Vazyme Biotech Co., Ltd.) was used to generate p35S::*LbHLH* via homologous recombination. p35S::*LbHLH* was introduced into *Agrobacterium tumefaciens* GV3101 cells, which were used to transform Arabidopsis. After screening for three generations with herbicides (0.1%, v/v), the Col-35S::*LbHLH* overexpression lines were subjected to physiological measurements.

Three Col-35S::*LbHLH* overexpression lines were selected for physiological characterization based on *LbHLH* expression (low, medium, and high). Specifically, positive transgenic plants were first identified using primers *LbHLH* OEAt-S and *LbHLH* OEAt-A based on the genomic sequences of the transgenic lines. mRNA was then extracted from different Col-35S::*LbHLH* lines using a FastPure Plant Total RNA Isolation kit (Vazyme Biotech Co., Ltd.) according to the manufacturer’s instructions. *LbHLH* expression levels in different Col-35S::*LbHLH* lines were analyzed by qRT-PCR using primers *LbHLH* RT-S and *LbHLH* RT-A (Table S1). Given that no homologs of *LbHLH* were detected in Arabidopsis, the line with the lowest *LbHLH* expression level (OE35) was used as a control (relative expression level set to 1) to calculate the expression levels of *LbHLH* in the Col-35S::*LbHLH* lines. Three biological replicates were performed for each group. Lines with high (OE40), medium (OE26), and low expression (OE4) levels were retained for analysis.

### Phenotypic observation and expression analysis of trichome/root hair-related genes in Col-35S::*LbHLH*

Trichome and root hair development were measured in three overexpression lines (OE4, OE26, and OE40) and the wild type. The trichomes on the first true leaves of one-week-old seedlings were counted under a dissecting microscope (Nikon, Japan). The root hairs 0.5 cm–1.5 cm from the tip of the roots of 5-day-old seedlings were counted. Trichome and root hair numbers were calculated with ImageJ software. Twenty seedlings were examined per line.

RNA was extracted from seedlings grown on 1/2MS medium for one week. The expression levels of ten genes involved in trichome differentiation and root hair fate determination were identified by qRT-PCR, including *AtTTG1*, *AtTRY*, *AtCPC*, *AtEGL3*, *AtGL1*, *AtGL3*, *AtSAD2*, *GLABRA 2* (*AtGL2*), *MYB DOMAIN PROTEIN 23* (*AtMYB23*), and *ZINC FINGER PROTEIN 5* (*AtZFP5*). The expression levels of genes related to root hair development, including the root hair initiation genes *ROOT HAIR DEFECTIVE 6* (*AtRHD6*) and *RING FINGER OF SEED LONGEVITY 1* (*AtRSL1*) and the root hair elongation gene *LJRHL1-LIKE 1* (*AtLRL1*) were also measured by qRT-PCR. All primers used in qRT-PCR are listed in Table S1 as gene name-RT-Sense (gene name-RT-S) and gene name-RT-Antisense (gene name-RT-A) (e.g., *AtEGL3*-RT-S and *AtEGL*3-RT-A). *AtActin* (amplified with primers *Atactin*-RT-S and *Atactin*-RT-A) was used as an internal control [23]; three replicate biological experiments were performed.

### Yeast two-hybrid assay to examine self-activation of LbHLH and identify candidate LbHLH-interacting proteins

The full-length CDS of *LbHLH* was cloned into pGBKT7 (BD) to generate BD-*LbHLH* using an NdeI digestion site by homologous recombination using a ClonExpress® II One Step Cloning Kit (Vazyme Biotech Co., Ltd.) and the primers BD-*LbHLH*-S and BD-*LbHLH*-A (Table S1). The same method was used to construct AD-*AtGL1* and AD-*AtGL3* using pGADT7 (AD) and primers AD-*AtGL1*-S, AD-*AtGL1*-A, AD-*AtGL3*-S, and AD-*AtGL3*-A with NdeI digestion sites (Table S1).

Seven groups were designed to test interactions between LbHLH and AtGL1 or AtGL3: BD-53&AD-T (positive control), BD-Lam&AD-T (negative control), BD&AD-AtGL1 (to verify AtGL1 self-activation), BD&AD-AtGL3 (to verify AtGL3 self-activation), BD-LbHLH&AD (to verify LbHLH self-activation), BD-LbHLH&AD-AtGL1 (to verify the interaction between LbHLH and AtGL1), and BD-LbHLH&AD-AtGL3 (to verify the interaction between LbHLH and AtGL3). The plasmids were transformed into Y2H Gold yeast (*Saccharomyces cerevisiae*) cells separately using Yeastmaker Yeast Transformation System 2 (Clontech Code No. 630439) following the manufacturer’s instructions. All groups were first cultured on SD/-Leu/-Trp medium to determine successful transformation based on the presence of colonies. Further interaction experiments were performed on both SD/-Leu/-Trp/X-a-gal/Aba (200 ng/ml) and SD/-Ade/-His/-Leu/-Trp/X-a-gal/Aba (200 ng/ml) media. The self-activation of LbHLH and verification of possible interactions were determined based on the growth status and blue color of colonies after 2 days of culture.

### Measuring *LbHLH* expression in *L. bicolor* during a time course of NaCl treatment, salt tolerance indices, and physiological indicators

*L. bicolor* seedlings were cultured in soil for 20 days and treated with 100 mM NaCl for 0, 12, 24, 48, and 72 h to generate samples for time course analysis. Total RNA was extracted from each sample and used for qRT-PCR with primers *LbHLH*-RT-S and *LbHLH*-RT-A (Table S1) to analyze the expression pattern of *LbHLH* over a time course of 100 mM NaCl treatment.

To investigate salt resistance among different transgenic lines, OE4, OE26, OE40, and wild-type (WT) seeds were germinated on 1/2MS medium (containing 1% agar) with different concentrations of NaCl (0, 50, 100, and 150 mM). The germinated seeds were counted each day for five days: a seed containing a radicle > 1 mm long that had emerged from the seed coat was considered to be germinated. The germination percentage (%) was calculated as the number of germinated seeds / total number of seeds × 100%. Thirty seeds were sown per line for each treatment, and three biological replicates were performed. The emergence of green cotyledons was used as an indicator of cotyledon growth. The cotyledon growth rate of each line was measured after three days of germination. Cotyledon growth rate (%) = (number of seeds with cotyledons / number of all tested seeds) × 100%.

At the same time, in order to avoid the influence of uneven germination trends at different NaCl treatments, seeds of all lines were first germinated on the 1/2MS basic medium for 4 days before transferred to different NaCl treatments (0, 50, 100, and 150 mM) for 5 days. All seedlings were photographed and the root lengths of different lines were measured using ImageJ software. Thirty replicates were performed per treatment. All seedlings were transplanted into the soil matrix and were continuously cultured in different NaCl treatments until bolting to see the effect of NaCl on the older plants.

To investigate the influences of NaCl on physiological indicators, the seedlings growing on 1/2MS medium for 5 days were transplanted into the soil matrix. After one week of adaptation, the seedlings were treated with 100 mM NaCl (NaCl dissolved in Hoagland solution, pH 6.2). After one week of treatment, the leaves of 20-day-old seedlings (0.5 g) were harvested separately. The Na^+^, K^+^, proline, and MDA (Malondialdehyde) contents were measured as described previously [30, 31]. Ion concentrations were measured with a flame photometer (Cole-Parmer, USA). Five replicates per measurement were performed for each line.

To verify the effect of *LbHLH* on alleviating salt stress, all lines were cultured in 180 mM sorbitol (causing the same osmotic pressure as 100 mM NaCl) and on 10 mM LiCl medium (without osmotic stress). After five days of culture, the germination rate was determined. Thirty seeds were sown per line for each treatment, and three biological replicates were performed. At the same time, all seeds were first germinated on the 1/2MS basic medium for 4 days before transferred to different treatments (180 mM sorbitol and 10 mM LiCl) for 5 days to measure the root lengths of different lines using ImageJ software. Thirty replicates were performed per treatment. Meanwhile, the growth of seedlings were photographed to compare the effects of ion stress and osmotic stress on different lines.

### qRT-PCR of marker genes related to salt stress in transgenic Arabidopsis

To investigate the expression of genes under salt stress, all lines were cultured for ~ 10 days in 1/2MS medium containing 0 or 100 mM NaCl and subjected to RNA extraction using a FastPure Plant Total RNA Isolation kit (RC401-01; Vazyme Biotech Co., Ltd.). The RNA was reverse-transcribed into cDNA and used for qRT-PCR.

Four marker genes involved in stress resistance were selected for qRT-PCR analysis: *SALT OVERLY SENSITIVE 1* (*AtSOS1*)*, AtSOS3, DELTA1-PYRROLINE-5-CARBOXYLATE SYNTHASE 1* (*AtP5CS1*)*,* and *AtP5CS2* (Table S1). *AtACTIN* was used as the internal control. Three biological replicates were performed.

### Statistical analysis

Statistical significance at *P *= 0.05 (Duncan’s multiple range tests) was determined using SPSS. ANOVA with orthogonal contrasts and mean comparison procedures was used to detect significant differences between treatments.

## Results

### Bioinformatics analysis and in situ hybridization of *LbHLH*

We cloned *LbHLH* based on its full-length sequence in the transcriptome data [19]. *LbHLH* contains a 2,067-bp open reading frame and encodes a 688-amino-acid protein (Figure S1A). LbHLH harbors a typical helix-loop-helix (HLH) domain between amino acids 480 and 526 and three low-complexity regions (Figure S1B). No gene sharing more than 30% similarity with *LbHLH* was detected by NCBI-BLAST analysis (Figure S1C).

**Fig. 1:**
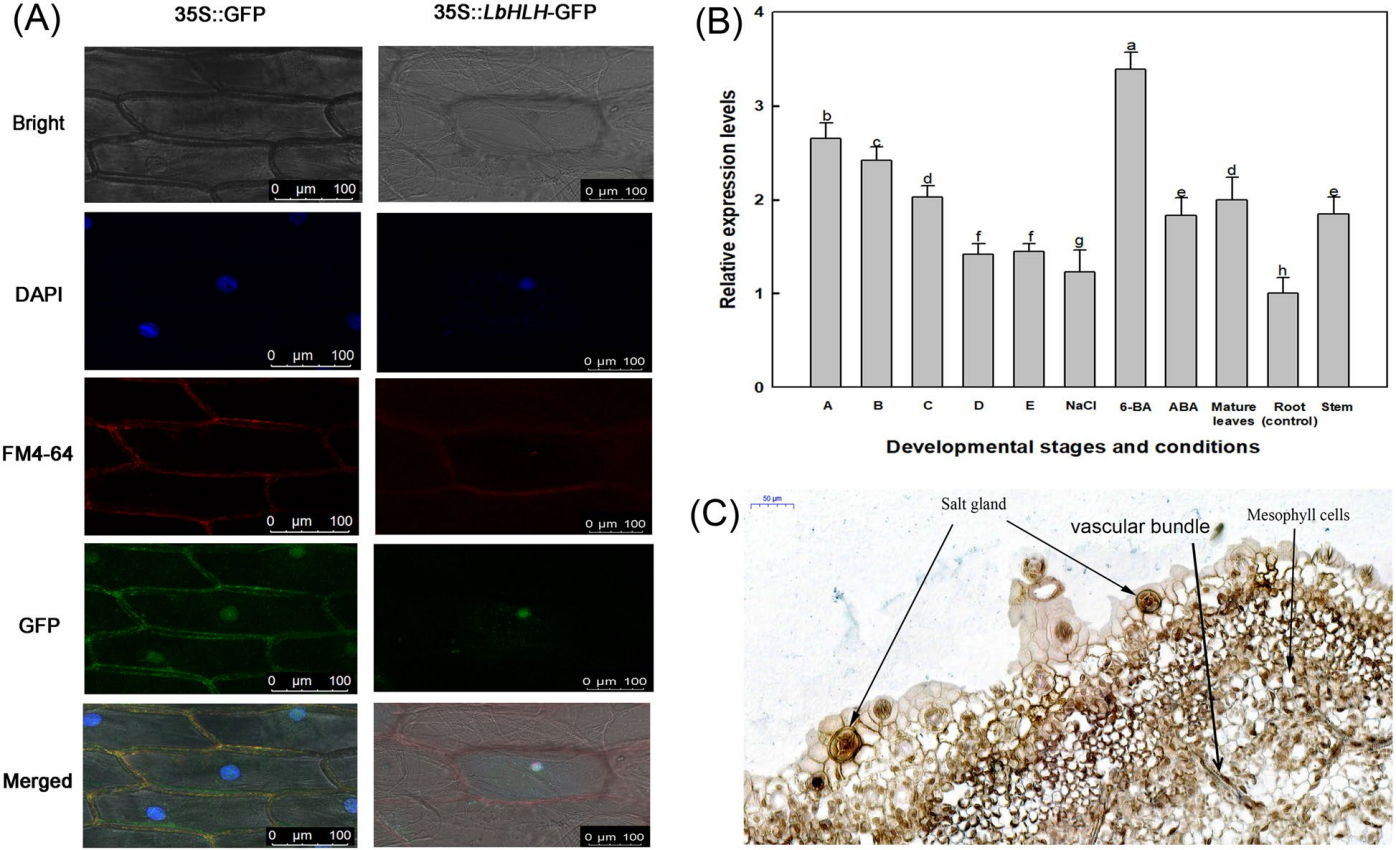
Subcellular localization and expression analysis of *LbHLH* in *L. bicolor*. **A** Subcellular localization analysis of 35S::LbHLH-GFP in onion epidermal cells. The GFP-LbHLH fusion protein was expressed in the nucleus. 35S::GFP was used as the empty vector control. Bar = 50 μm. DAPI appears as blue fluorescence specifically in the nucleus, and FM4-64 appears as red fluorescence at the plasma membrane. **B ***LbHLH* expression levels in leaves at different developmental stages and under different treatments. A: stage A, undifferentiated, 4–5 days after sowing; B: stage B, salt gland differentiation, 6–7 days after sowing; C: stage C, stomatal differentiation, 8–10 days after sowing; D: stage D, epidermal differentiation, 11–16 days after sowing; E: stage E, mature, more than 17 days after sowing; Mature leaves: fully expanded leaves; NaCl: mature leaves from stage E seedlings after 24 h of 200 mM NaCl treatment; 6-BA: mature leaves from stage E seedlings grown on 0.04 mg/L 6-BA; ABA: mature leaves from stage E seedlings grown on 0.1 mg/L ABA. Roots and stems were collected from stage E seedlings. Data are the means ± SD of three replicates; different letters indicate significant differences at *P* = 0.05 according to Duncan’s multiple range test. **C** In situ hybridization of *LbHLH* using developing leaves in *L. bicolor*. *LbHLH* transcripts were detected using a digoxin-labeled anti-sense probe, which produces a blue-violet color

To determine the subcellular localization of LbHLH, we transformed onion epidermal cells with *Agrobacterium tumefaciens* carrying p35S::*LbHLH*-GFP. As shown in Fig. 1A, compared to the positions of DAPI and FM4-64 staining, *LbHLH*-GFP was located only in the nucleus, while unfused GFP was located in the nucleus and plasma membrane. We also analyzed the expression pattern of *LbHLH* in *L. bicolor* at different developmental stages and under different treatments (Fig. 1B). *LbHLH* was expressed at the highest level in stage A leaves and at the lowest level in roots. *LbHLH* expression was highly induced by 6-BA treatment.

Given that *LbHLH* was highly expressed during early salt gland development, we performed in situ hybridization to determine whether LbHLH localizes to salt glands in *L. bicolor*. Hybridization signals were detected in all salt glands, partial mesophyll cells and vascular bundle (Fig. 1C), suggesting that *LbHLH* may functions in salt gland development and differentiation.

### Analysis of the *LbHLH* promoter and histochemical localization of LbHLH

**Fig. 2:**
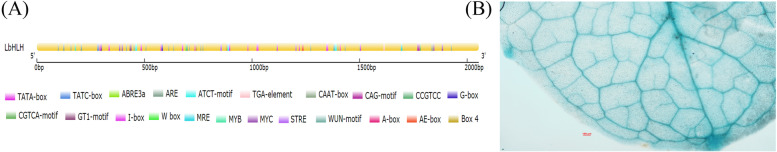
Analysis of the elements and activity of the *LbHLH* promoter. **A** Elements in the *LbHLH* promoter predicted using PlantCARE; the map was drawn using CSDS 2.0. Different colors represent different elements, as indicated by the key below the map. **B** Analysis of *LbHLH* promoter activity in young Arabidopsis seedlings transformed with p*LbHLH*::GUS. GUS staining (blue) is seen mainly in the veins

Further localization of LbHLH was verified in Arabidopsis. We identified the 2,055 bp promoter sequence of *LbHLH* based on its sequence in the *L. bicolor* genome. As shown in Fig. 2A, the *LbHLH* promoter is enriched in typical TATA box and CAAT box elements and harbors various stress-responsive elements such as ABRE and ARE. The identification of MYB binding sites suggests that *LbHLH* might be regulated by MYB-type transcription factors. To verify the site of *LbHLH* expression, we generated p*LbHLH*::GUS and transformed Arabidopsis with this construct. Analysis of GUS staining patterns revealed that *LbHLH* is expressed in leaf veins in Arabidopsis (Fig. 2B).

### *LbHLH* participates in trichome and root hair development in Arabidopsis

**Fig. 3:**
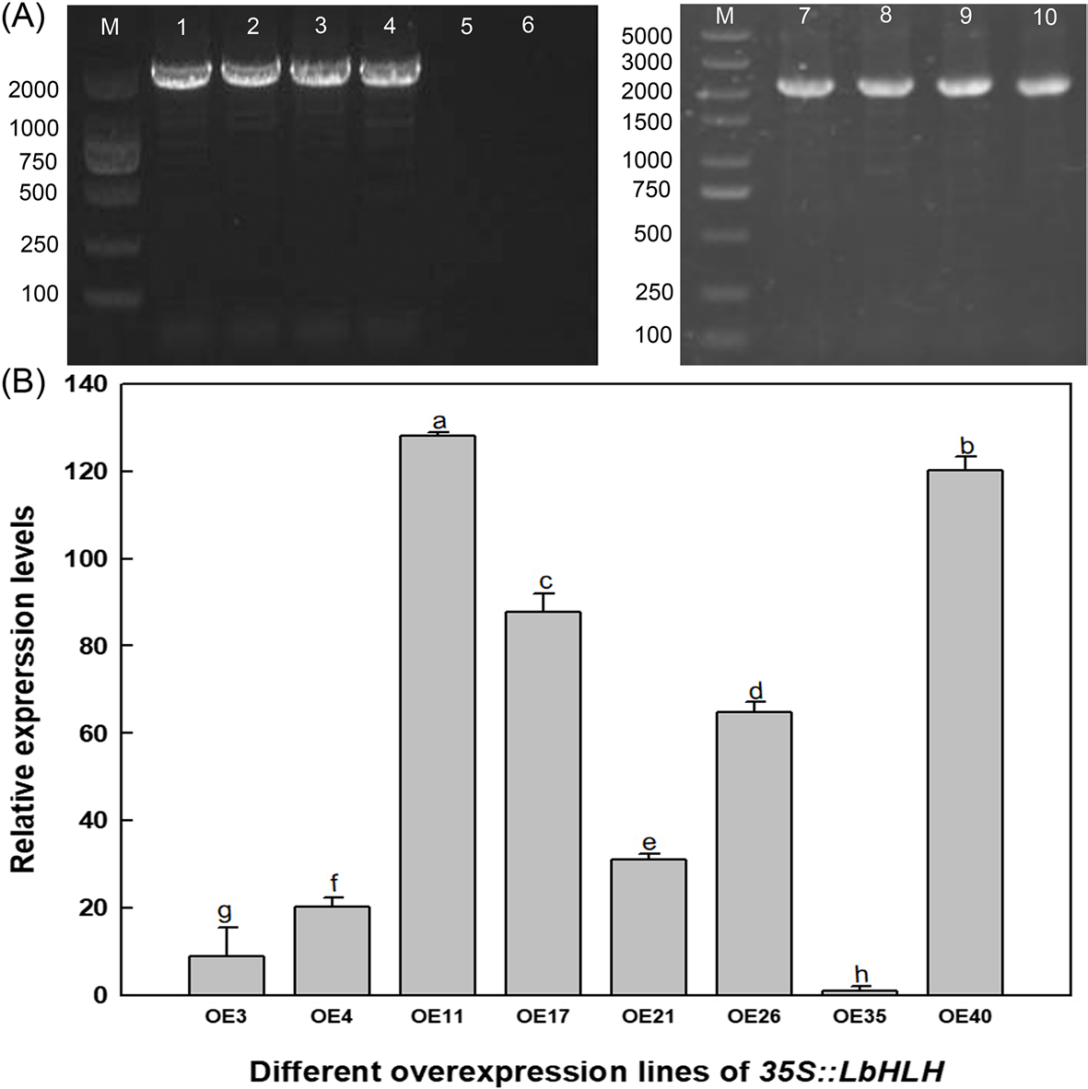
Identification and analysis of Col-35S::*LbHLH* plants. **A** PCR of genomic DNA from the Col 35S::*LbHLH* lines; lanes 1–4 and 7–10, different transgenic lines; lane 5, blank control with ddH_2_O used as a template; lane 6, negative control with wild-type DNA used as a template. These two pictures were cut from Figure S2 and Figure S3. **B** Expression levels of *LbHLH* in Col 35S::*LbHLH* examined by qRT-PCR. Data are means ± SD of three replicates; different letters indicate significant differences at *P* = 0.05 according to Duncan’s multiple range test

To explore the role of *LbHLH* in trichome and root hair formation, we heterologously expressed *LbHLH* in Arabidopsis ecotype Col-0. Eight lines harboring Col-35S::*LbHLH* (Fig. 3A) were identified and their gene expression levels analyzed by qRT-PCR (Fig. 3B). Lines OE4, OE26, and OE40, with relative low, medium, and high *LbHLH* expression levels, respectively, were selected for phenotypic observation.

**Fig. 4:**
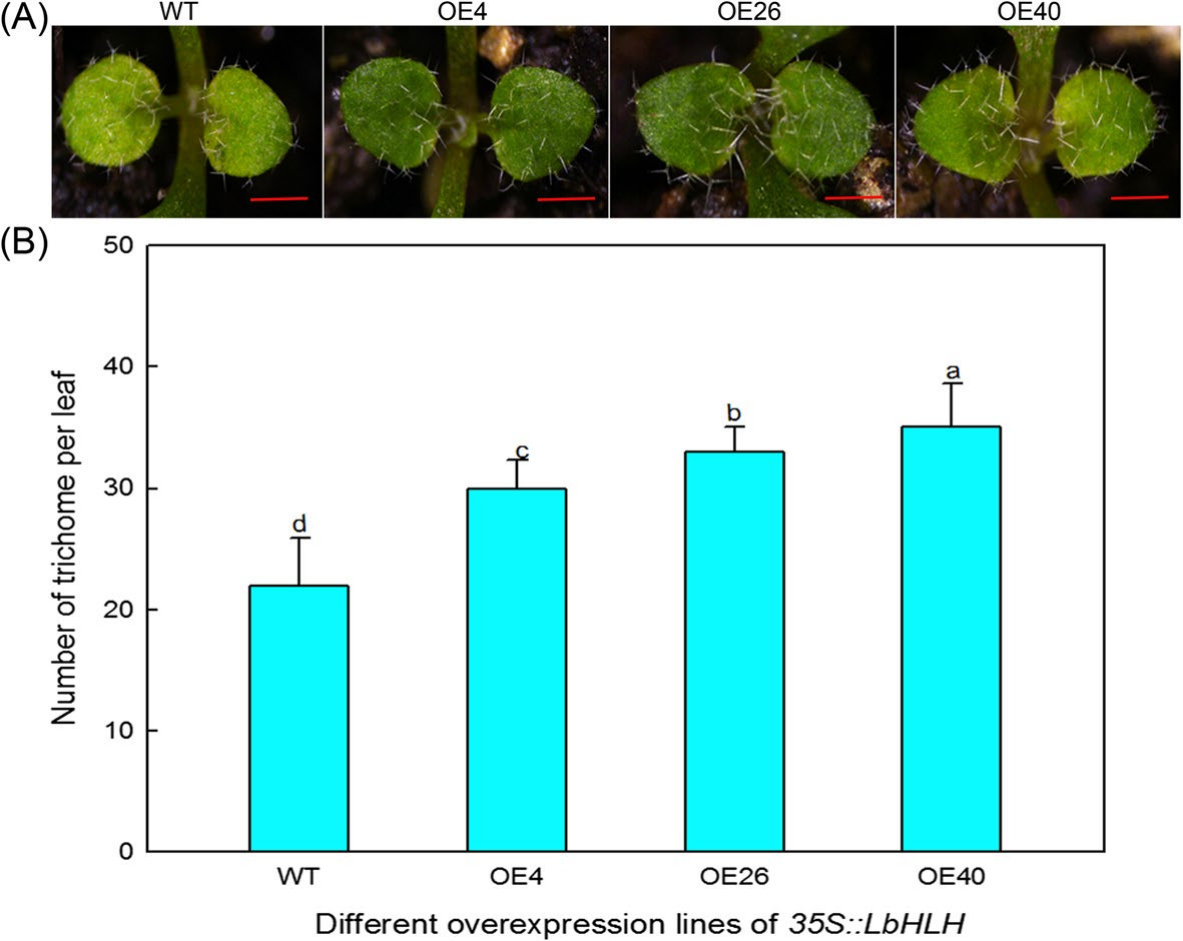
Analysis of trichomes of Col-35S::*LbHLH* plants. **A** Trichomes on the first two rosette leaves of Col-0 wild-type (WT) and Col 35S::*LbHLH* (OE4, OE26, and OE40) plants. Photographs show 2-week-old soil-grown seedlings. **B** Number of trichomes on the first two rosette leaves of WT, OE4, OE26, and OE40 plants. Data are mean ± SD of 20 plants; different letters indicate significant differences at *P* = 0.05 according to Duncan’s multiple range test

We compared the number of trichomes on the first true leaves of wild type (WT), OE4, OE26, and OE40, and found that the Col-35S::*LbHLH* overexpression lines contained significantly more trichomes than the WT (Fig. 4A). In addition, the expression level of *LbHLH* had a dose effect on the number of trichomes (Fig. 4B), that is, the higher the expression level of *LbHLH*, the more trichomes that were produced. These results indicate that *LbHLH* promotes trichome development.

**Fig. 5:**
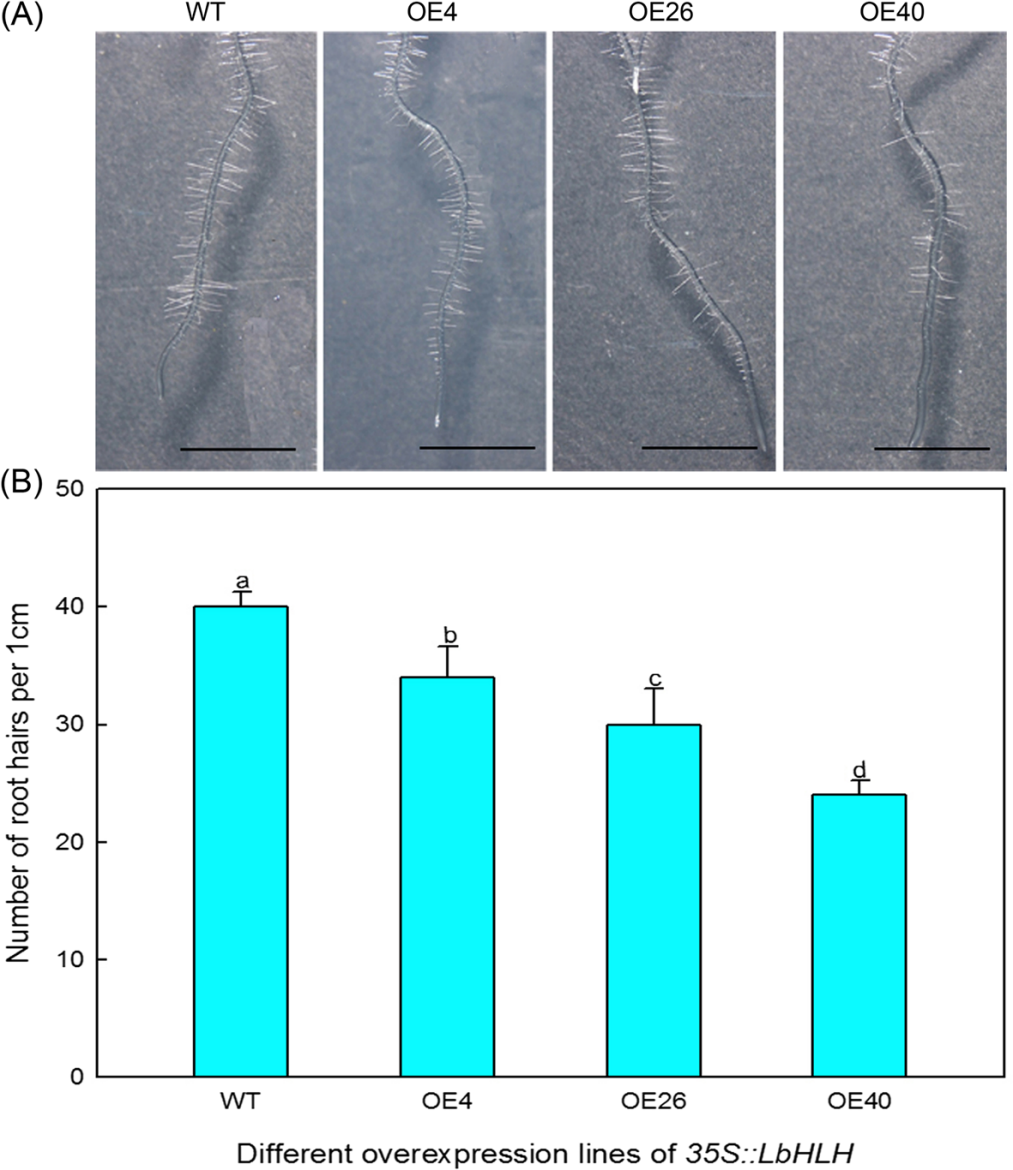
Analysis of root hairs in Col-35S::*LbHLH* plants. **A** Phenotypes of root hairs in WT and Col 35S::*LbHLH* (OE4, OE26, and OE40) plants after 5 days of culture on 1/2MS medium. **B** A 1-cm segment of each root (0.5–1.5 cm from the root tip) was chosen to measure root hair number from 20 plants per line. Data are mean ± SD of 20 plants; different letters indicate significant differences at *P* = 0.05 according to Duncan’s multiple range test

The same genes are involved in the initiation of trichome and root hair development, but they play opposite roles in these processes. Therefore, we counted the root hairs in each line. The Col-35S::*LbHLH* overexpression lines produced fewer roots hairs than the WT (Fig. 5A), and this phenotype also showed a dose effect, as the number of root hairs decreased with increasing *LbHLH* expression (Fig. 5B). These results indicate that LbHLH has an inhibitory effect on root hair development.

### LbHLH interacts with AtGL1 to interfere with root hair development

**Fig. 6:**
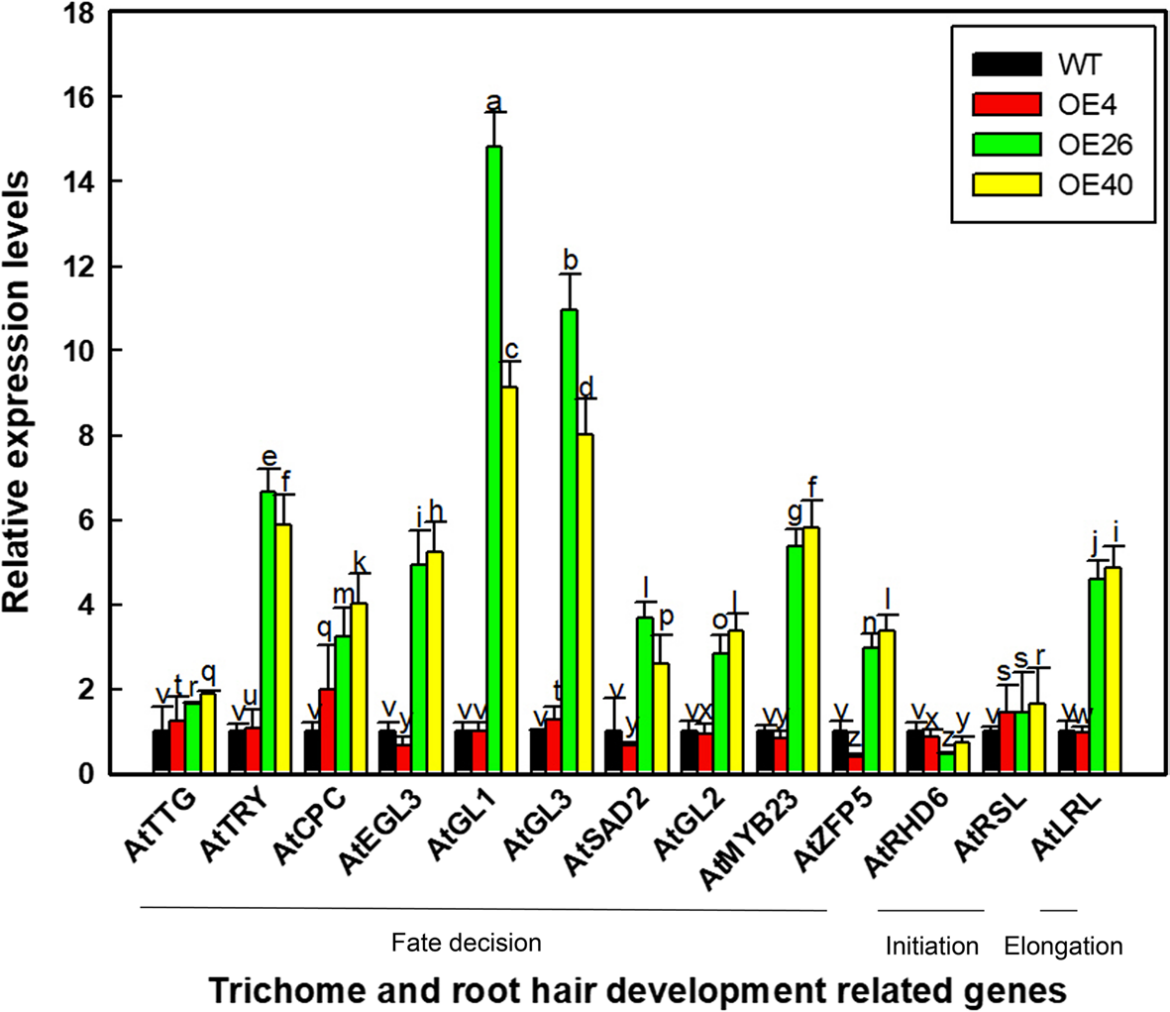
Analysis of phenotype-related gene expression. qRT-PCR analysis of the expression levels of genes involved in root hair and trichome development, including ten fate decision genes (*AtTTG1*, *AtTRY*, *AtCPC*, *AtEGL3*, *AtGL1*, *AtGL3*, *AtSAD2*, *AtGL2*, *AtMYB23*, and *AtZFP5*), two root hair initiation genes (*AtRHD6* and *AtRSL1*), and one root hair elongation gene (*AtLRL1*) in five-day-old seedlings. Data are means ± SD of three biological replicates; different letters indicate significant differences at *P* = 0.05 according to Duncan’s multiple range test

Given that the heterologous expression of *LbHLH* increased the number of trichomes and reduced the number of root hairs in Arabidopsis, we analyzed the expression levels of genes related to trichome and root hair initiation and development in these lines, including *AtTTG1*, *AtTRY*, *AtCPC*, *AtEGL3*, *AtGL1*, *AtGL3*, *AtSAD2*, *AtGL2*, *AtMYB23*, *AtLRL1*, *AtRHD6*, *AtRSL1*, and *AtZFP5* (Fig. 6). Most genes were expressed at the highest levels in OE40, whereas no significant difference in expression was detected between the WT and OE4. Among these genes, *AtGL1* and *AtGL3* were the most highly induced in OE26 and OE40 vs. the WT. Therefore, LbHLH most likely interacts with AtGL1 or AtGL3 to influence trichome and root hair development.

**Fig. 7:**
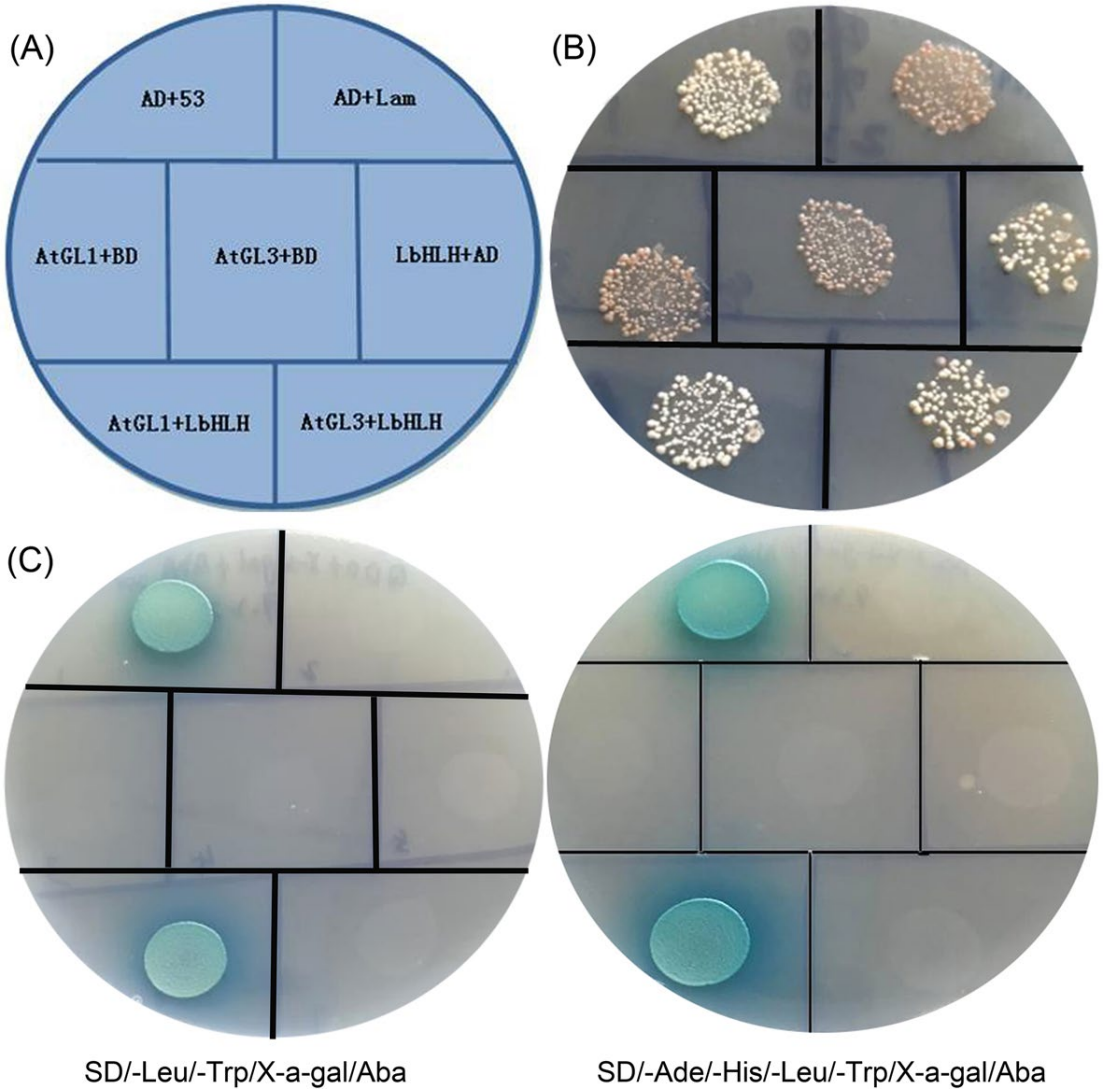
Yeast two-hybrid assay of the interaction between LbHLH and AtGL1 or AtGL3. **A** Verification of self-activation and interactions in Y2H Gold yeast. AD + 53: AD-T + BD-53 (positive control); AD + Lam: AD-T + BD-Lam (negative control); AtGL1 + BD: AD-AtGL1 + BD (test of AtGL1 self-activation); AtGL3 + BD: AD-AtGL3 + BD (test of AtGL3 self-activation); LbHLH + AD: BD-LbHLH + AD (test of LbHLH self-activation); AtGL1 + LbHLH: AD-AtGL1 + BD-LbHLH (test of Interaction 1); AtGL3 + LbHLH: AD-AtGL3 + BD-LbHLH (test of Interaction 2). **B** Yeast transformation on SD/-Leu/-Trp medium. **C** Tests of self-activation and interactions on SD/-Leu/-Trp/X-a-gal/Aba (200 ng/ml) and SD/-Ade/-His/-Leu/-Trp/X-a-gal/Aba (200 ng/ml) media

To test for interactions between LbHLH and AtGL1 or LbHLH and AtGL3 in vitro, we performed a yeast two-hybrid assay (Fig. 7A). All colonies grew normally on SD/-Leu/-Trp medium, indicating that the yeast transformation was successful (Fig. 7B). When grown on SD/-Leu/-Trp/X-a-gal/Aba and SD/-Ade/-His/-Leu/-Trp/X-a-gal/Aba media, only the positive control and BD-*LbHLH*&AD-AtGL1 turned blue and showed normal growth (Fig. 7C), whereas BD-*LbHLH*&AD-AtGL3 produced no signal. No self-activation of LbHLH was detected. These results indicate that LbHLH strongly interacts with AtGL1, an MYB-like protein required for root hair development, which could explain why root hair formation was significantly inhibited in the transgenic lines.

### *LbHLH* is induced by salt stress in *L. bicolor* and significantly increases the salt resistance of Arabidopsis during germination

**Fig. 8:**
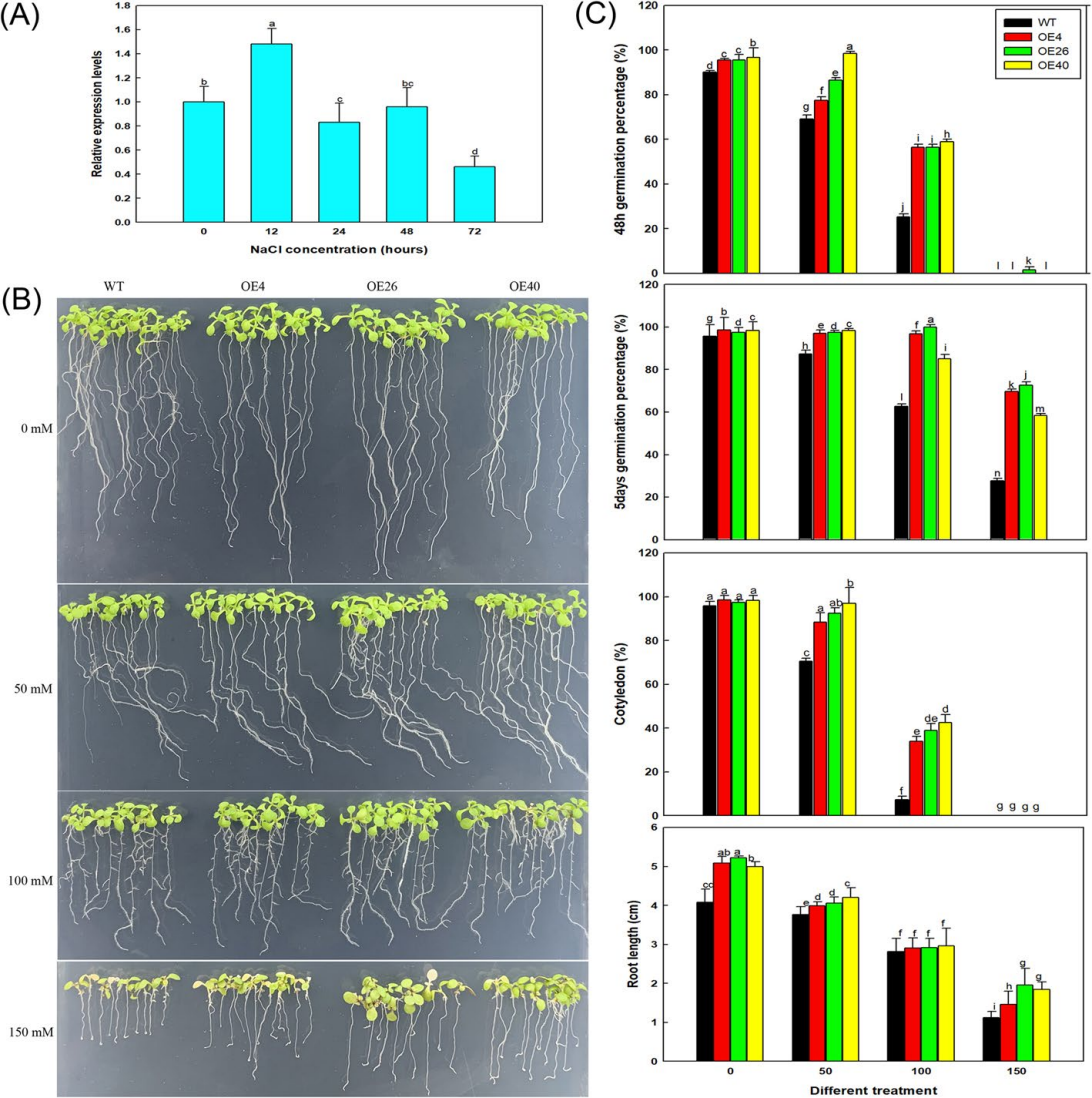
Col 35S::*LbHLH* lines exhibit increased salt tolerance. **A** The expression patterns of *LbHLH* in *L. bicolor* after 100 mM NaCl treatment for different periods of time (0, 12, 24, 48, and 72 h); the 0 h NaCl treatment was used as the control (relative expression level = 1). **B** Seeds of all lines were first germinated on the 1/2MS basic medium for 4 days before transferred to different NaCl treatments (0, 50, 100, and 150 mM) for 5 days. **C** Analysis of germination and seedling growth under different NaCl treatments. Germination percentage was measured at 48 h and 5 days after sowing. Thirty seeds per line were sown per treatment, and three replicates were performed. Germination percentage data are the mean ± SD of three replicates. The cotyledon growth rate (expressed as the percentage of plants with emerged cotyledons) was calculated 3 days after sowing on different media. Thirty seeds per line were sown per treatment, and three replicates were performed. Cotyledon growth rate data are mean ± SD of three replicates. Root lengths of nine-day-old seedlings of (**B**) were determined using ImageJ software. Thirty seedlings were analyzed per line. Root length data are the mean ± SD of 30 plants. Different letters indicate significant differences at *P* = 0.05 according to Duncan’s multiple range test

Since the heterologous expression of *LbHLH* reduced the number of root hairs in Arabidopsis, we wondered whether this phenotype is related to salt tolerance. We therefore examined the expression pattern of *LbHLH* in *L. bicolor* over a time course of a 100 mM NaCl treatment (Fig. 8A). Compared to the level of *LbHLH* expression the start of the NaCl treatment, expression was significantly increased after 12 h of NaCl treatment but returned to a lower level after 24 h. These results suggest that the expression of *LbHLH* can be induced by transient NaCl.

We next examined the salt tolerance of the transgenic Arabidopsis lines during germination and early plant growth by sowing seeds of each line on medium containing a gradient of NaCl concentrations. As expected, the transgenic lines showed better growth than the wild type under high-salt conditions (Fig. 8B), and OE40 (with the highest *LbHLH* expression level) grew the best. Compared with WT, all three Col-35S::*LbHLH* lines showed a higher germination percentage at 48 h after sowing (Fig. 8C), especially under the 100 mM NaCl treatment, where the germination rates of the OE lines were more than twice that of the WT. The effect was even more apparent under the 150 mM NaCl treatment, as only a few OE26 seeds germinated, compared to none for the other lines. After 5 days of culture, there were no obvious differences among the lines under the 0 and 50 mM NaCl treatments; however, under the 100 and 150 mM NaCl treatments, the germination percentage was significantly higher in the transgenic lines than in the WT (Fig. 8C).

The growth rates of cotyledons showed a similar trend (Fig. 8C). Under the 50 and 100 mM NaCl treatments, cotyledon growth was much better in the Col-35S::*LbHLH* lines than in the WT. Root growth was inhibited with increasing NaCl concentration, but at each NaCl concentration, the roots of the Col-35S::*LbHLH* lines were longer than WT roots. This phenomenon is especially obvious when treated with 150 mM NaCl (Fig. 8C). Further culturing of older plants under gradient NaCl treatments showed that the overexpression lines had the typical better growth than WT (Figure S2).

**Fig. 9:**
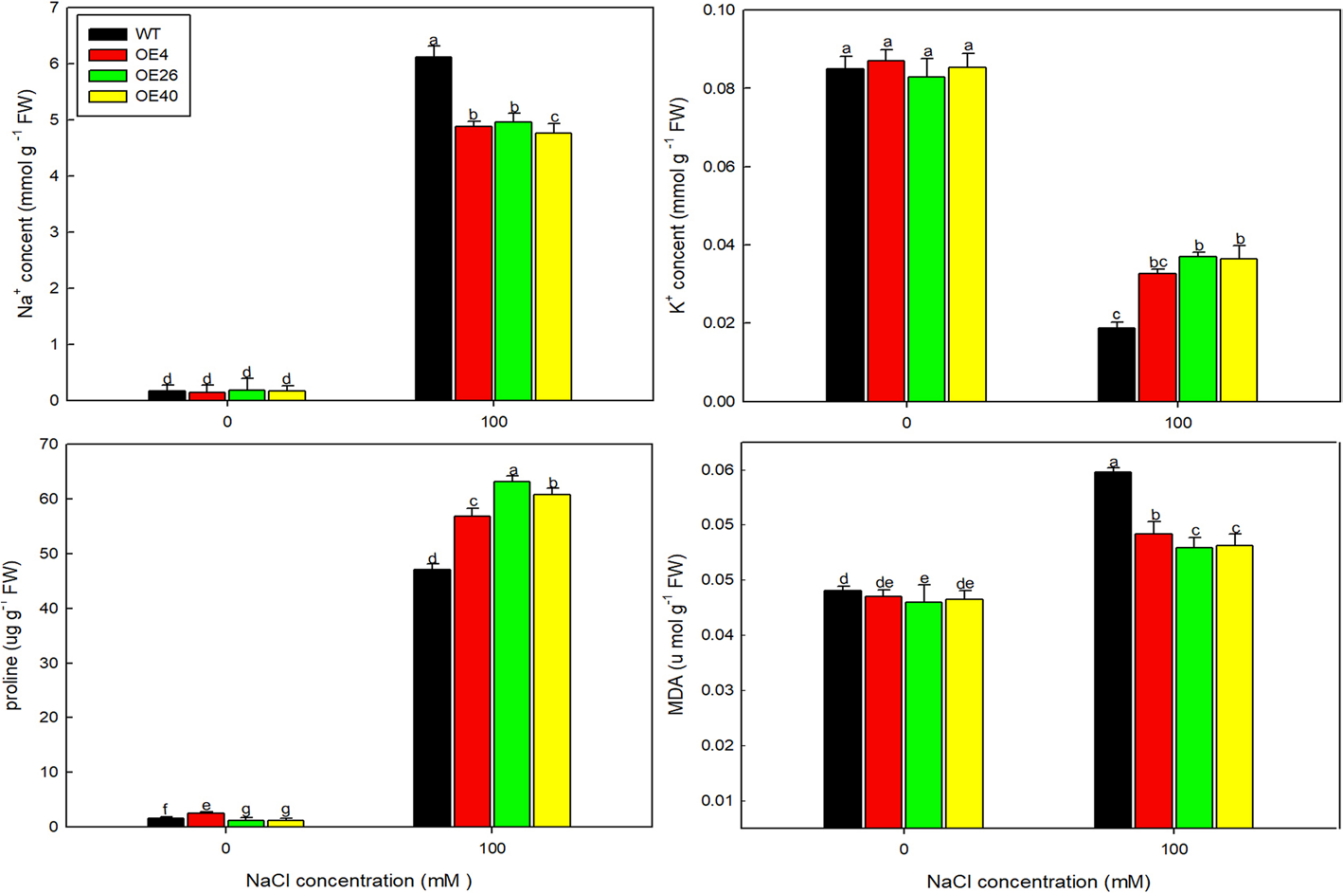
Na^+^, K^+^, MDA, and proline contents of Col 35S::*LbHLH* lines under control and 100 mM NaCl growth conditions. Measurements were performed on two-week-old seedlings in soil, and five replicates were performed per line. Data are the mean ± SD of five plants; different letters indicate significant differences at *P* = 0.05 according to Duncan’s multiple range test

To determine the possible reason for the enhanced salt resistance of the transgenic lines, we measured various physiological indicators in plants under the 0 and 100 mM NaCl treatments (Fig. 9). OE40 accumulated the least amount of Na^+^ and the most proline and K^+^ under 100 mM NaCl conditions, while the opposite results were obtained for the WT. MDA is the final decomposition product of membrane lipid peroxidation, and its content can reflect the degree of stress injury of plants. MDA contents were lower in the transgenic lines than in the WT under NaCl treatment, indicating that the overexpression lines suffered less injury than the WT under salt treatment.

### *LbHLH* enhances salt tolerance by enhancing the ability to resist osmotic stress

**Fig. 10:**
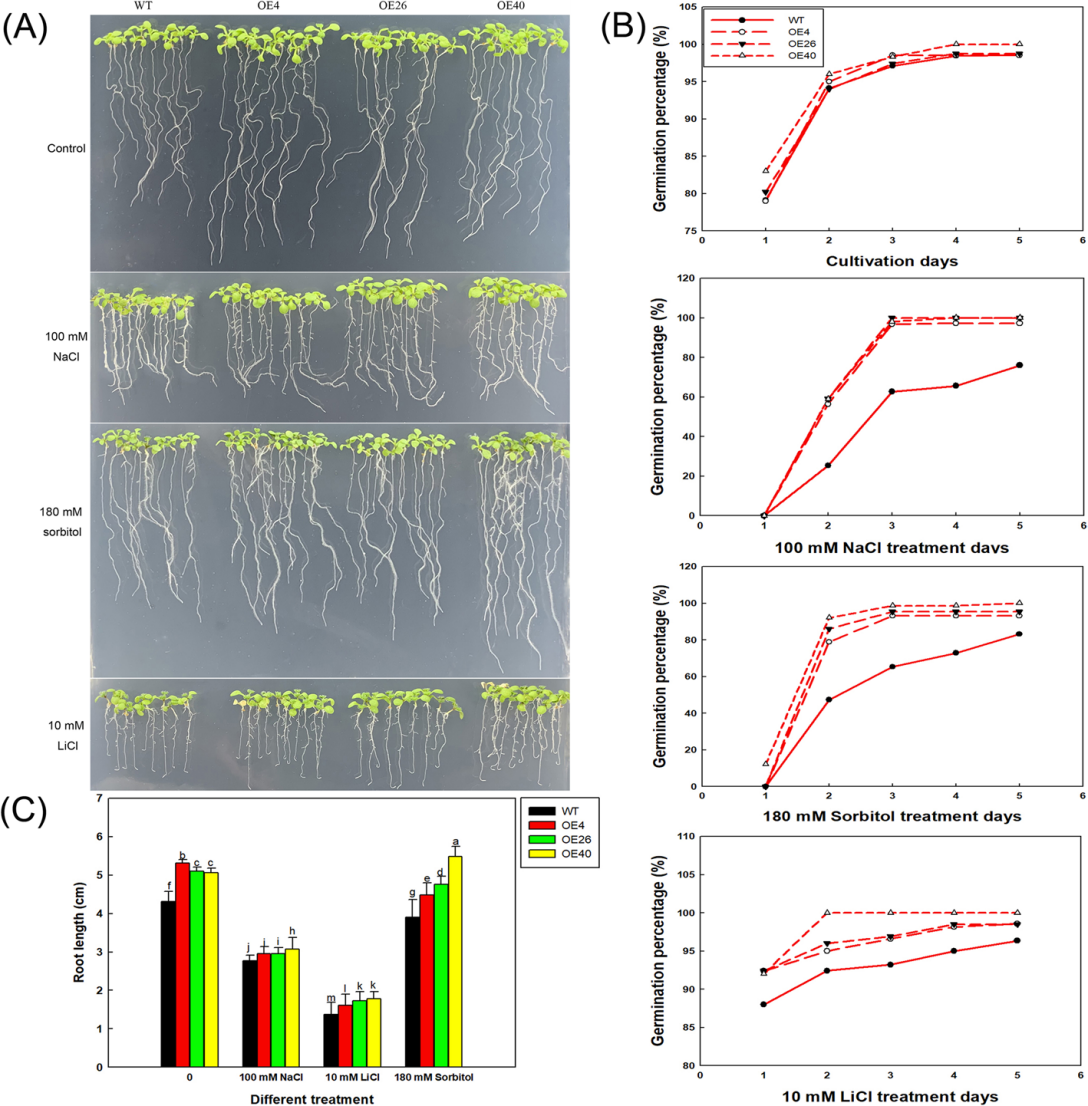
*LbHLH* enhances salt tolerance by enhancing the ability to resist osmotic stress. **A** The seeds of all lines were first germinated on the 1/2MS basic medium for 4 days before transferred to different media containing 180 mM sorbitol, 10 mM LiCl, or 100 mM NaCl for 5 days. **B** Germination percentage calculated each day after sowing on different media. Thirty seeds per line were sown per treatment, and three replicates were performed. Data are the mean ± SD of three replicates. **C** Root lengths of (A) were determined using ImageJ software. Thirty seedlings were analyzed per line. Data are the mean ± SD of 30 plants; different letters indicate significant differences at *P* = 0.05 according to Duncan’s multiple range test

To further explore how *LbHLH* improves salt tolerance, we cultured the three transgenic Arabidopsis lines and the WT in medium containing 180 mM sorbitol, which has the same osmotic potential as 100 mM NaCl, and in medium containing 10 mM LiCl, which induces the same ionic stress as 100 mM NaCl (Fig. 10A). Under the 10 mM LiCl treatment, all lines showed similar levels of growth inhibition, and no growth advantage was detected in the transgenic lines. However, under isotonic sorbitol treatment, the overexpression lines showed the same trends in growth as they did under 100 mM NaCl treatment, i.e., OE40 had the highest germination rate (Fig. 10B) and the longest root (Fig. 10C). It is worth mentioning that compared with the control treatment, OE40 almost did not suffer from osmotic stress. These results indicate that WT plants suffered both osmotic and ionic stress under NaCl treatment, whereas the heterologous expression of *LbHLH* significantly improved resistance to osmotic stress, allowing the transgenic lines to perform better than WT at the germination stage.

**Fig. 11:**
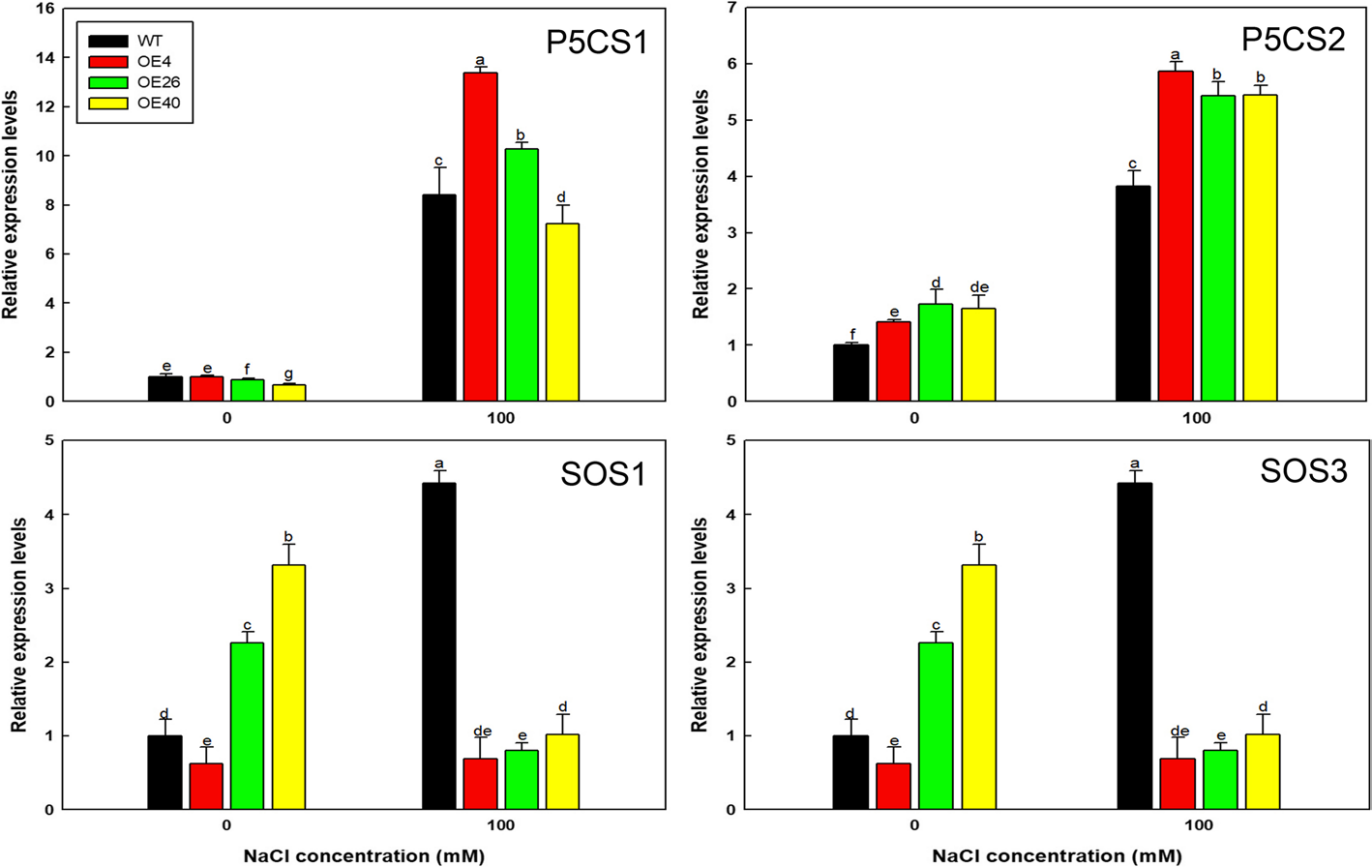
Relative expression levels of *AtSOS1*, *AtSOS3*, *AtP5CS1*, and *AtP5CS2*. The expression level of each gene was measured by qRT-PCR with three biological replicates (separate experiments); different letters indicate significant differences at *P* = 0.05 according to Duncan’s multiple range test

Finally, to explore why transformation with *LbHLH* significantly improved salt tolerance in Arabidopsis at the molecular level, we examined the expression of marker genes in these plants under salt stress. *AtP5CS1* and *AtP5CS2* were expressed at much higher levels in the Col-35S::*LbHLH* lines than in the WT, while the expression of *AtSOS1* and *AtSOS3* declined in the transgenic lines (Fig. 11); these expression levels corresponded to the levels of proline accumulation (Fig. 9), osmotic stress resistance in these plants (Fig. 10) and the low accumulation of Na^+^ in shoots.

## Discussion

*L. bicolor* is a typical recretohalophyte with salt glands. Increasing numbers of genes in *L. bicolor* have been shown to participate in salt resistance, but all of these genes are homologs of genes of known function, such as *LbTTG1*, *LbTRY*, and *LbSAD2*. No genome sequences of plant species with salt glands are currently available. Therefore, identifying the activities of genes of unknown function that are expressed during salt gland development may be crucial for understanding salt gland development and salt resistance. Here, we demonstrated that LbHLH, which was annotated to be a protein of unknown function, induces the expression of genes related to trichome and root hair development by interacting with AtGL1 in Arabidopsis. Furthermore, LbHLH improves salt tolerance, primarily by enhancing osmotic resistance due to high NaCl levels.

Bioinformatic analysis showed that LbHLH contains an HLH (helix-loop-helix) domain. HLH domains are primarily detected in transcription factors such as OrbHLH2O in Arabidopsis and SbHLH148 in rice [32, 33]. Surprisingly, in the current study, we determined that LbHLH has no self-activation activity (Fig. 7). In situ hybridization showed that *LbHLH* is expressed in the salt glands of *L. bicolor*. Therefore, we suggest that LbHLH interacts with other transcription factors or functional proteins in the halophyte *L. bicolor* to regulate salt gland development. Moreover, in situ hybridization signal was detected not only in salt gland but also vascular bundle (Fig. 1C), and further histochemical staining driven by LbHLH promoter in Arabidopsis also find high expression in leaf veins (Fig. 2B). Given that lignin may lead to the autofluorescence of salt gland under UV excitation [16], there may be some common differentiation mechanism between salt gland and vascular bundle from the aspect of lignin based on the position evidences.

In addition, the heterologous expression of *LbHLH* led to an increase in trichome formation in Arabidopsis. Based on previously reported transcriptomes of *L. bicolor* at different stages of development [19], salt gland and trichome development might involve homologous genes with similar developmental patterns of expression. These findings strongly suggest that *LbHLH* may be related to salt gland development, and they explain the why of heterologous expression of *LbHLH* affects trichome development in Arabidopsis.

The interaction between LbHLH and AtGL1 was detected in vitro in a yeast two-hybrid assay. AtGL1 positively regulates trichome development in Arabidopsis [34, 35], and the *gl1* mutant lacks trichomes on its leaf surfaces. These findings suggest that LbHLH regulates trichome and root hair development by directly interacting with AtGL1. Interestingly, this gene has opposite effects on the development of aboveground trichomes and underground root hairs; that is, *LbHLH* promotes trichome development and inhibits root hair growth via the same interaction with AtGL1. Why *LbHLH* plays opposite roles in trichome and root hair development is still unknown. Nevertheless, other genes (AtTTG1, ATGL1, AtTRY, AtCPC, et al.)[36] have been shown to play opposite roles in trichome and root hair initiation; the underlying mechanism would be worth investigating in the future. Furthermore, opposite effects of LbHLH on root hair and trichome development are also detected under salt treatment (data not shown), that is to say, trichome of transgenic lines was still promoted and root hair was inhibited under NaCl treatment, indicating that the effect of LbHLH on the epidermal structure differentiation was not eliminated by NaCl. Furthermore, the reduced root hair can help to reduce the Na^+^ absorption.

The reduced root hair contribute to the less Na^+^ absorption under NaCl treatment because root hairs directly participate in the absorption of ions. As expected, the Col-35S::*LbHLH* lines showed much better germination than the WT. The reduced number of root hairs makes the Col-35S::*LbHLH* lines absorb less Na^+^, suffer less ionic stress, and accumulate less MDA than the WT, phenotypes that were also observed in Arabidopsis expressing *LbTTG1* [22]. Moreover, a typical dose effect was observed in the transgenic lines: the stronger the expression of *LbHLH*, the greater the salt tolerance of the lines. What’ more, SOS1 was considered the key Na^+^/H^+^ exchanger in Na^+^ exclusion [37, 38] to avoid stress to organelles, but SOS1 showed down-regulated in the transgenic lines compared with WT under NaCl treatment (Fig. 11), which may be related to the low Na^+^ accumulation due to the low absorption by reduced root hairs in the transgenic lines. Moreover, there are also evidences that SOS1 also plays a significant role in xylem Na^+^ loading to confer salt tolerance [39–41], so it is speculated that LbHLH overexpression also affected SOS1-mediated release of Na^+^ into the transpiration flow and delivery to the shoot.

Given that the reduction in the root hair may compromise plant’s ability to take up mineral ions, such as less Na^+^ absorption in transgenic lines, but unexpectedly the content of K^+^ was increased, which may be explained by more K^+^ retention in the shoot resulting from lower Na^+^ loading. Furthermore, K^+^ was considered the inorganic osmolytes [42] and more accumulation in transgenic lines can significantly enhance the salt tolerance. Sorbitol and LiCl are widely used to simulate osmotic and ionic stress, respectively, and the effects of these substances can easily be determined based on phenotype [30]. The Col-35S::*LbHLH* lines showed enhanced tolerance to sorbitol but not LiCl, indicating that the increased tolerance to NaCl stress in the transgenic lines was due to increased tolerance of osmotic stress. In *L. bicolor*, *LbHLH* expression was highly induced after 12 h of NaCl treatment, suggesting that this gene responds to short-term osmotic stress and may contribute to salt tolerance in this halophyte.

The current study examined the function of *LbHLH* mainly by heterologous expression of the gene in the model plant Arabidopsis. However, in situ hybridization and expression analysis in *L. bicolor* during development strongly suggested that *LbHLH* may function in salt gland development. Given that a transformation system has been developed for *L. bicolor* [18], it should be possible to use CRISPR-mediated gene editing to further investigate the function of *LbHLH* in *L. bicolor* and its role in salt gland development. Here, we demonstrated that the function of an unknown gene in *L. bicolor* could be successfully studied, laying the foundation for studying the roles of salt glands in salt resistance and the utilization of saline soils in the future.

## Conclusion

LbHLH of *L. bicolor*, which was annotated to be a protein of unknown function positioned in salt gland by in situ hybridization, had highest expression at early leaf development stage. Overexpression of LbHLH induces the expression of genes related to trichome and root hair development by interacting with AtGL1 in Arabidopsis. Furthermore, LbHLH improves salt tolerance, primarily by reducing root hair development and enhancing osmotic resistance due to high NaCl levels.

## Supplementary Information

**Additional file 1: Figure S1 **Bioinformatic analysis of *LbHLH*. **(A)** Nucleotide and deduced amino acid sequences of *LbHLH* analyzed by DNAMAN. **(B)** Conserved domains of *LbHLH*, including a HLH domain located at amino acids 480-526, drawn with SMART. **(C)** Similarity (%) between *LbHLH* and the most closely related genes from other species detected by NCBI-BLAST analysis. All genes share <30% similarity with *LbHLH*. **Figure S2 **The older plants of all lines under gradient NaCl treatments cultured for one month. **Figure S3 **The original gel image of Figure 3 (A-left). **Figure S4 **The original gel image of Fig. 3 (A-right). **Table S1** The primers used in this paper.

## Data Availability

The datasets used and/or analysed during the current study available from the corresponding author on reasonable request.

## Abbreviations

HLH: Helix-Loop-Helix
*GL1*: *GLABRA1*
*TTG1*: *TRANSPARENT TESTA GLABRA1*
*GL3*: *GLABRA3*
*EGL3*: *ENHANCER OF GLABRA 3*
*SAD2*: *SUPER SENSITIVE TO ABA AND DROUGHT2*
*TRY*: *TRIPTYCHON*
*CPC*: *CAPRICE*
*AtGL2*: *GLABRA 2*
*AtMYB23*: *MYB DOMAIN PROTEIN 23*
*AtZFP5*: *ZINC FINGER PROTEIN 5*
*AtRHD6*: *ROOT HAIR DEFECTIVE 6*
*AtRSL1*: *RING FINGER OF SEED LONGEVITY 1*
*AtLRL1*: *LJRHL1-LIKE 1*
*AtSOS1*: *SALT OVERLY SENSITIVE 1*
*AtP5CS1*: *DELTA1-PYRROLINE-5-CARBOXYLATE SYNTHASE 1*
Col-0: Columbia-0
qPCR: Quantitative PCR
CDS: Coding sequence
BD: pGBKT7
AD: pGADT7
WT: Wild-type

## References

[1] Munns R, Tester M (2008). Mechanisms of salinity tolerance. Annu Rev Plant Biol.

[2] Munns R (2005). Genes and salt tolerance: bringing them together. New Phytol.

[3] Shabala S, Wu H, Bose J (2015). Salt stress sensing and early signalling events in plant roots: current knowledge and hypothesis. Plant Sci.

[4] Flowers TJ, Colmer TD (2008). Salinity tolerance in halophytes. New Phytol.

[5] Asano T, Hayashi N, Kobayashi M, Aoki N, Miyao A, Mitsuhara I, Ichikawa H, Komatsu S, Hirochika H, Kikuchi S (2012). A rice calcium-dependent protein kinase OsCPK12 oppositely modulates salt-stress tolerance and blast disease resistance. Plant J.

[6] Li Z, Li G, Qin P (2010). The prediction of ecological potential for developing salt-tolerant oil plants on coastal saline land in Sheyang Saltern China. Ecol Eng.

[7] de Paz JM, de Paz JM, Visconti F, Zapata R, Sánchez J (2004). Integration of two simple models in a geographical information system to evaluate salinization risk in irrigated land of the Valencian Community Spain. Soil Use Manage.

[8] Song J, Wang B (2015). Using euhalophytes to understand salt tolerance and to develop saline agriculture: Suaeda salsa as a promising model. Ann Bot.

[9] Guo J, Lu C, Zhao F, Gao S, Wang B (2020). Improved reproductive growth of euhalophyte Suaeda salsa under salinity is correlated with altered phytohormone biosynthesis and signal transduction. Funct Plant Biol.

[10] Guo J, Dong X, Li Y, Wang B (2020). NaCl treatment markedly enhanced pollen viability and pollen preservation time of euhalophyte Suaeda salsa via up regulation of pollen development-related genes. J Plant Res.

[11] Yuan F, Guo J, Shabala S, Wang B (1954). Reproductive Physiology of Halophytes: Current Standing. Front Plant Sci.

[12] Yuan F, Leng B, Wang B (2016). Progress in Studying Salt Secretion from the Salt Glands in Recretohalophytes: How Do Plants Secrete Salt?. Front Plant Sci.

[13] Feng Z, Sun Q, Deng Y, Sun S, Zhang J, Wang B (2014). Study on pathway and characteristics of ion secretion of salt glands of Limonium bicolor. Acta Physiol Plant.

[14] Morris L, Yun K, Rutter A, Zeeb BA (2019). Characterization of Excreted Salt from the Recretohalophytes *Distichlis spicata* and *Spartina pectinata*. J Environ Qual.

[15] Lu C, Feng Z, Yuan F, Han G, Guo J, Chen M, Wang B: The SNARE protein LbSYP61 participates in salt secretion in *Limonium bicolor*. Environ Exper Bot 2020;176:104076.

[16] Deng Y, Feng Z, Yuan F, Guo J, Suo S, Wang B (2015). Identification and functional analysis of the autofluorescent substance in Limonium bicolor salt glands. Plant Physiol Biochem.

[17] Ding F, Chen M, Sui N, Wang BS (2010). Ca2+ significantly enhanced development and salt-secretion rate of salt glands of Limonium bicolor under NaCl treatment. S Afr J Bot.

[18] Yuan F, Chen M, Yang J, Leng B, Wang B (2014). A system for the transformation and regeneration of the recretohalophyte Limonium bicolor. Vitro Cellular Dev Biol Plant.

[19] Yuan F, Lyu MJ, Leng BY, Zheng GY, Feng ZT, Li PH, Zhu XG, Wang BS (2015). Comparative transcriptome analysis of developmental stages of the Limonium bicolor leaf generates insights into salt gland differentiation. Plant Cell Environ.

[20] Yuan F, Lyu MJ, Leng BY, Zhu XG, Wang BS (2016). The transcriptome of NaCl-treated Limonium bicolor leaves reveals the genes controlling salt secretion of salt gland. Plant Mol Biol.

[21] Xu Y, Jiao X, Wang X, Zhang H, Wang B, Yuan F: Importin-β From the Recretohalophyte *Limonium bicolor* Enhances Salt Tolerance in *Arabidopsis thaliana* by Reducing Root Hair Development and Abscisic Acid Sensitivity. Front Plant Sci 2021;11:582459. 10.3389/fpls.2020.582459 PMC7838111 33519843

[22] Yuan F, Leng B, Zhang H, Wang X, Han G, Wang B (2019). A WD40-repeat protein from the recretohalophyte limonium bicolor enhances trichome formation and salt tolerance in Arabidopsis. Front Plant Sci.

[23] Leng B, Wang X, Yuan F, Zhang H, Lu C, Chen M, Wang B (2021). Heterologous expression of the Limonium bicolor MYB transcription factor LbTRY in Arabidopsis thaliana increases salt sensitivity by modifying root hair development and osmotic homeostasis. Plant Sci.

[24] Bao J, Sha S, Zhang S (2011). Changes in germinability, lipid peroxidation, and antioxidant enzyme activities in pear stock (Pyrus betulaefolia Bge.) seeds during room- and low-temperature storage. Acta Physiologiae Plantarum.

[25] Sui N, Tian S, Wang W, Wang M, Fan H (2017). Overexpression of Glycerol-3-Phosphate Acyltransferase from Suaeda salsa improves salt tolerance in Arabidopsis. Front Plant Sci.

[26] Liang AX, Han L, Hua GH, Geng LY, Sang L, Liu XB, Guo AZ, Yang LG (2009). Construction of a fusion protein expression vector pGS/2SS-M4GFP without antibiotic resistance gene and its subcellular localization in different cell lines. Biologicals.

[27] Sun W, Cao Z, Li Y, Zhao Y, Zhang H: A simple and effective method for protein subcellular localization using Agrobacterium-mediated transformation of onion epidermal cells. Biologia. 2007;62(5):529–32.

[28] Ng LC, Ramduny D, Airey JA, Singer CA, Keller PS, Shen XM, Tian H, Valencik M, Hume JR. Orai1 interacts with STIM1 and mediates capacitative Ca^2+^ entry in mouse pulmonary arterial smooth muscle cells. Am J Physiol Cell Physiol. 2010;299(5):C1079–1090. 10.1152/ajpcell.00548.2009 PMC2980318 20739625

[29] Amnuaykanjanasin A, Epstein L (2006). A class Vb chitin synthase in Colletotrichum graminicola is localized in the growing tips of multiple cell types, in nascent septa, and during septum conversion to an end wall after hyphal breakage. Protoplasma.

[30] Han G, Yuan F, Guo J, Zhang Y, Sui N, Wang B (2019). AtSIZ1 improves salt tolerance by maintaining ionic homeostasis and osmotic balance in Arabidopsis. Plant Sci.

[31] Guo J, Li Y, Han G, Song J, Wang B (2018). NaCl markedly improved the reproductive capacity of the euhalophyte Suaeda salsa. Funct Plant Biol.

[32] Seo JS, Joo J, Kim MJ, Kim YK, Nahm BH, Song SI, Cheong JJ, Lee JS, Kim JK, Choi YD (2011). OsbHLH148, a basic helix-loop-helix protein, interacts with OsJAZ proteins in a jasmonate signaling pathway leading to drought tolerance in rice. Plant J.

[33] Zhou J, Li F, Wang JL, Ma Y, Chong K, Xu YY (2009). Basic helix-loop-helix transcription factor from wild rice (OrbHLH2) improves tolerance to salt- and osmotic stress in Arabidopsis. J Plant Physiol.

[34] Bloomer RH, Juenger TE, Symonds VV (2012). Natural variation in GL1 and its effects on trichome density in Arabidopsis thaliana. Mol Ecol.

[35] Eckstein A, Grzyb J, Hermanowicz P, Labuz J, Banas AK (2019). A role for GLABRA1 in dark-induced senescence. Acta Biochim Pol.

[36] Yang C, Ye Z (2013). Trichomes as models for studying plant cell differentiation. Cell Mol Life Sci.

[37] Qiu QS, Guo Y, Dietrich MA, Schumaker KS, Zhu JK (2002). Regulation of SOS1, a plasma membrane Na^+^/H^+^ exchanger in *Arabidopsis thaliana*, by SOS2 and SOS3. Proc Natl Acad Sci.

[38] Mario BA, Zhu JK (2009). SIK1/SOS2 networks: decoding sodium signals via calcium-responsive protein kinase pathways. Pfluegers Arch.

[39] Yadav NS, Shukla PS, Jha A, Agarwal PK, Jha B (2012). The SbSOS1 gene from the extreme halophyte *Salicornia brachiata* enhances Na^+^ loading in xylem and confers salt tolerance in transgenic tobacco. BMC Plant Biol.

[40] Zhu M, Shabala L, Cuin TA, Huang X, Zhou M, Munns R, Shabala S (2016). Nax loci affect SOS1-like Na^+^/H^+^ exchanger expression and activity in wheat. J Exp Bot.

[41] Zhu M, Zhou M, Shabala L, Shabala S (2017). Physiological and molecular mechanisms mediating xylem Na^+^ loading in barley in the context of salinity stress tolerance. Plant Cell Environ.

[42] Zhu M, Zhou M, Shabala L, Shabala S (2015). Linking osmotic adjustment and stomatal characteristics with salinity stress tolerance in contrasting barley accessions. Funct Plant Biol.

