# Supplementary material for: A novel gene LbHLH from the halophyte Limonium bicolor enhances salt tolerance via reducing root hair development and enhancing osmotic resistance
Source: BMC Plant Biol. 2021 Jun 22;21:284. doi: 10.1186/s12870-021-03094-3 (PMC8218485; doi:10.1186/s12870-021-03094-3)
Supplement: Supplementary file 1 — Additional file 1: Figure S1 Bioinformatic analysis of LbHLH. (A) Nucleotide and deduced amino acid sequences of LbHLH analyzed by DNAMAN. (B) Conserved domains of LbHLH, including a HLH domain located at amino acids 480-526, drawn with SMART. (C) Similarity (%) between LbHLH and the most closely related genes from other species detected by NCBI-BLAST analysis. All genes share <30% similarity with LbHLH. Figure S2 The older plants of all lines under gradient NaCl treatments cultured for one month. Figure S3 The original gel image of Figure 3 (A-left). Figure S4 The original gel image of Fig. 3 (A-right). Table S1 The primers used in this paper. [file 12870_2021_3094_MOESM1_ESM.docx]

**A novel gene** **LbHLH from the halophyte *Limonium bicolor* enhances salt tolerance via reducing root hair development and enhancing osmotic resistance**

Xi Wang, Yingli Zhou, Yanyu Xu, Baoshan Wang*, Fang Yuan*

Shandong Provincial Key Laboratory of Plant Stress, College of Life Sciences, Shandong Normal University, Ji’nan, Shandong, P.R. China

*Corresponding authors

E-mail:

Baoshan Wang: bswang@sdnu.edu.cn

Fang Yuan: yuanfang@sdnu.edu.cn

Tel.: 0086 531 86180197; Fax: 0086 531 86180197

**Running title:** LbHLH functions in salt resistance

Figure S1


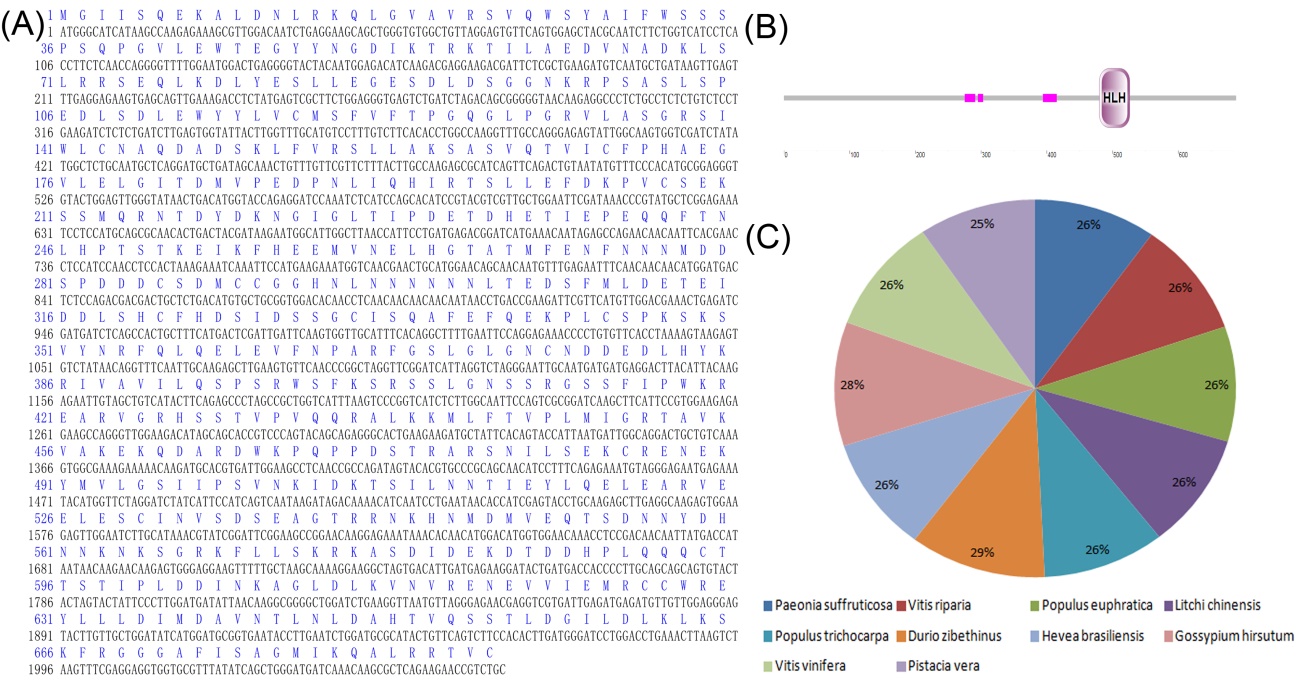


Figure S2


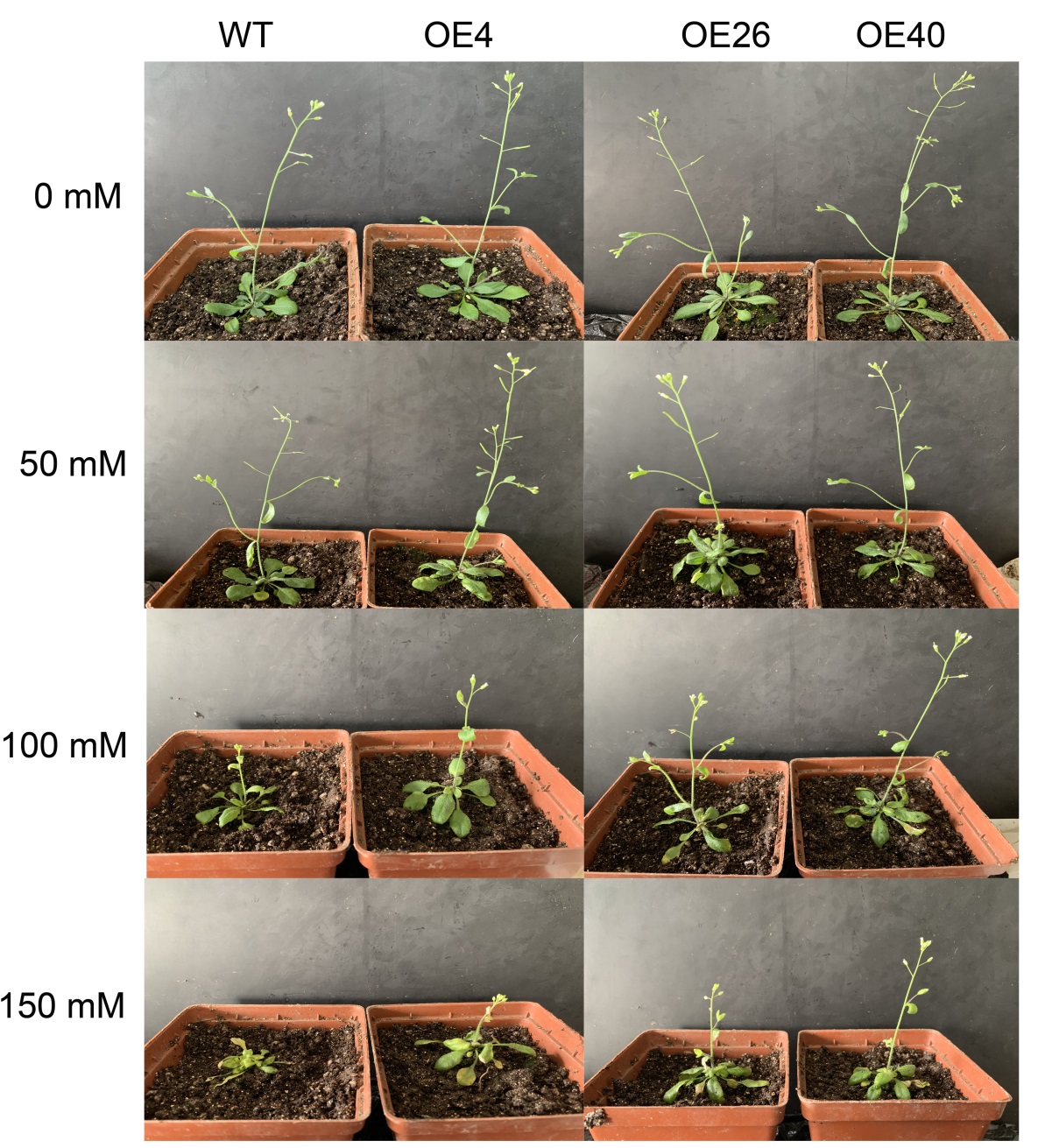


Figure S3


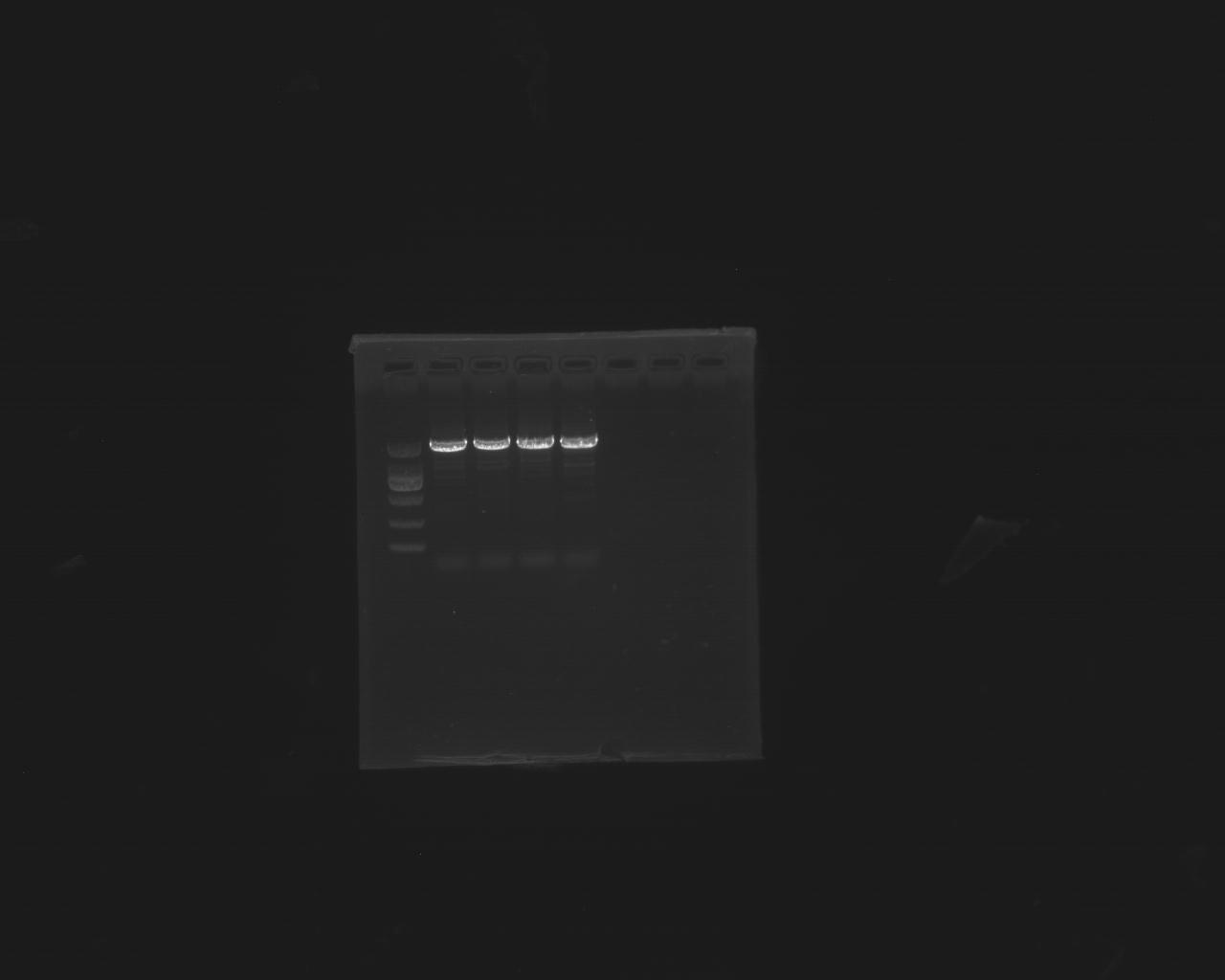


Figure S4


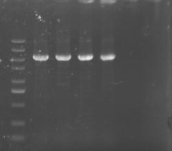


Table S1 The primers used in this paper

| **Name** | **Oligonucleotide sequence** | **Role** |
| --- | --- | --- |
| *LbHLH*-S | 5-TCCTTTTCTGGGGTTTGGGG-3 | Full length amplification |
| *LbHLH*-A | 5-GTCATCGCAACAGTCTCCAT-3 |  |
| *LbHLH* OE-S | 5-CGGGGATCCTCTAGAGTCGACATGGGCATCATAAGCCAAGAGA-3 | Construction of p35S::*LbHLH*-GFP vectors digested with SalⅠ |
| *LbHLH* OE-A | 5-GCCCTTGCTCACCATGTCGACGCAGACGGTTCTTCTGAGCG-3 |  |
| *LbHLH-*RT-S | 5-AGTGGAAGAGTTGGAATC-3 | Real-time PCR in different developmental stages, condition and Arabidopsis overexpression lines |
| *LbHLH-*RT-A | 5- TAATTGTTGTCGGAGGTT -3 |  |
| *Lbtubulin*-RT-S | 5-GGTTGAGTGAGCAGTTCAC-3 |  |
| *Lbtubulin*-RT-A | 5-GATAACCAGCCACACCTTAGC-3 |  |
| RT-*Atactin*-S | 5-GGTAACATTGTGCTCAGTGGTGG-3 |  |
| RT-*Atactin*-A | 5-AACGACCTTAATCTTCATGCTGC-3 |  |
| *LbHLH* OEAt-S | 5- ACGGGGGACTCTTGACCATGGATGGGCATCATAAGCCAAGAGA-3 | Construction of Col-35S::*LbHLH* and Col::*pLbHLH*-GUS |
| *LbHLH* OEAt-A  *LbHLH*-P-S  *LbHLH*-P-S  3301-*LbHLH*-P-S  3301-*LbHLH*-P-A | 5- TTACCCTCAGATCTACCATGGGCAGACGGTTCTTCTGAGCG-3  5- CTTTCTCTTGGCTTATGAT-3  5-CATTTCGTGCAACCTCAC-3  5-TGCAGGCATGCAAGCTtAGATTAAAGTTAATGCGCTCCTGC-3  5-CCTCAGATCTACCATGgTTCGTGCAACCTCACAAAATAGA-3 |  |
| *AtTRY*-RT-S | 5-CTTCTTCTTCTTGTTCGCTCTA-3 | RT-qPCR verification of trichome development and root hair fate decision genes in *OE-LbHLH* |
| *AtTRY*- RT-A | 5-ACGGTCAGTGTTATCCATTAC-3 |  |
| *AtCPC*- RT-S | 5-TCCGAAGAGGTGAGTAGT-3 |  |
| *AtCPC*- RT-A | 5-ACGAGTTTATACATCCGAGAA-3 |  |
| *AtTTG1*- RT-S | 5-TATTGAGAAGTCTGTTGT-3 |  |
| *AtTTG1*- RT-A | 5-ATTGTAGAATGTTCCTTATC-3 |  |
| *AtGL1*- RT-S | 5-CCTTCTTCTTGTCATCAT-3 |  |
| *AtGL1*- RT-A  *AtGL3*- RT-S  *AtGL3*- RT-A  *AtEGL3*- RT-S  *AtEGL3*- RT-A | 5-ATCATTAGTAGTTGCCATT-3  5-GCTTAGATGTGCTTGGAGAG-3  5-GAGGATTGAACCGAATGAGAA-3  5-AATCTTCTGGTCTGTCTC-3  5-AATCGTCTTCCTTGTCTT-3 |  |
| *AtSAD2*- RT-S  *AtSAD2*- RT-A  *AtGL2*- RT-S | 5-TGGAGCTCTTTGTGACAAATTG-3  5-GTGCAATGCTTTACGAAAGTTG-3  5-GACAGTGAAATCGAGAGA-3 |  |
| *AtGL2*- RT-A | 5-GTGACTCTACTCCATCAG-3 |  |
| *AtMYB23* - RT-S | 5-GACGAATCCAAACTCAAA-3 |  |
| *AtMYB23* - RT-A | 5-AGGCAATACCCATTAGTAA-3 |  |
| *AtZFP5*- RT-S | 5-AGAGGCTTCAGCTTCAAG-3 |  |
| *AtZFP5*- RT-A | 5-GACCAATCTTCGTTGTACAC-3 |  |
| *AtRHD6* - RT-S | 5-ACGAGAGTTATTGGAATCA-3 | RT-qPCR verification of root hair initation and elongation genes in *OE-LbHLH* |
| *AtRHD6*- RT-A | 5-TGCCAACTAAAGTGAGAG-3 |  |
| *AtRSL1* - RT-S | 5-CTTGGTTCTCATCACAAC-3 |  |
| *AtRSL1* - RT-A | 5-GGAACAACGGTGTAATATC-3 |  |
| *AtLRL1*- RT- S | 5-TTGCGGAAAGAATGAAAG-3 |  |
| *AtLRL1*- RT-A | 5-CCTTGACTTGGAGTTGTA-3 |  |
| *AtSOS1*- RT-S | 5-TTCATCATCCTCACAATGGCTCTAA-3 | RT-qPCR verification of salt tolerance related marker genes in *OE-LbHLH* |
| *AtSOS1*- RT-A | 5-CCCTCATCAAGCATCTCCCAGTA-3 |  |
| *AtSOS3*- RT-S | 5-AGAGGAAGATAGAGATGTAAGC-3 |  |
| *AtSOS3*- RT-A | 5-ATTATGTATGTGAGATGGAGAGT-3 |  |
| *AtP5CS1*- RT-S | 5-CAAGATGAGATTACATTCG-3 |  |
| *AtP5CS1*- RT-A | 5-GGTTATGATGACAGGAAT-3 |  |
| *AtP5CS2*- RT-S | 5-GTGACGGAAGATAGTGAA-3 |  |
| *AtP5CS2*- RT-A | 5-TCCTGCTTGTGCTTATTC-3 |  |
